# A pragmatic health centre-based evaluation comparing the effectiveness of a PCV13 schedule change from 3+0 to 2+1 in a high pneumococcal carriage and disease burden setting in Malawi: a study protocol

**DOI:** 10.1136/bmjopen-2021-050312

**Published:** 2021-06-17

**Authors:** Todd D Swarthout, Ana Ibarz-Pavon, Gift Kawalazira, George Sinjani, James Chirombo, Andrea Gori, Peter Chalusa, Farouck Bonomali, Roseline Nyirenda, Edwin Bulla, Comfort Brown, Jacquline Msefula, Marjory Banda, Jean Kachala, Charles Mwansambo, Marc YR Henrion, Stephen B Gordon, Neil French, Robert S Heyderman

**Affiliations:** 1 NIHR Global Health Research Unit on Mucosal Pathogens, Division of Infection and Immunity, University College London, London, UK; 2 Malawi-Liverpool-Wellcome Trust Clinical Research Programme, University of Malawi College of Medicine, Blantyre, Malawi; 3 Centre for Global Vaccine Research, Institute of Infection and Global Health, University of Liverpool Faculty of Health and Life Sciences, Liverpool, UK; 4 Ministry of Health, Lilongwe, Malawi; 5 Ministry of Education, Blantyre, Malawi; 6 Department of Clinical Sciences, Liverpool School of Tropical Medicine, Liverpool, UK

**Keywords:** epidemiology, epidemiology, public health, paediatric infectious disease & immunisation, public health

## Abstract

**Introduction:**

*Streptococcus pneumoniae* (the pneumococcus) is commonly carried as a commensal bacterium in the nasopharynx but can cause life-threatening disease. Transmission occurs by human respiratory droplets and interruption of this process provides herd immunity. A 2017 WHO Consultation on Optimisation of pneumococcal conjugate vaccines (PCV) Impact highlighted a substantial research gap in investigating why the impact of PCV vaccines in low-income countries has been lower than expected. Malawi introduced the 13-valent PCV (PCV13) into the national Expanded Programme of Immunisations in 2011, using a 3+0 (3 primary +0 booster doses) schedule. With evidence of greater impact of a 2+1 (2 primary +1 booster dose) schedule in other settings, including South Africa, Malawi’s National Immunisations Technical Advisory Group is seeking evidence of adequate superiority of a 2+1 schedule to inform vaccine policy.

**Methods:**

A pragmatic health centre-based evaluation comparing impact of a PCV13 schedule change from 3+0 to 2+1 in Blantyre district, Malawi. Twenty government health centres will be randomly selected, with ten implementing a 2+1 and 10 to continue with the 3+0 schedule. Health centres implementing 3+0 will serve as the direct comparator in evaluating 2+1 providing superior direct and indirect protection against pneumococcal carriage. Pneumococcal carriage surveys will evaluate carriage prevalence among children 15–24 months, randomised at household level, and schoolgoers 5–10 years of age, randomly selected from school registers. Carriage surveys will be conducted 18 and 33 months following 2+1 implementation.

**Analysis:**

The primary endpoint is powered to detect an effect size of 50% reduction in vaccine serotype (VT) carriage among vaccinated children 15–24 months old, expecting a 14% and 7% VT carriage prevalence in the 3+0 and 2+1 arms, respectively.

**Ethics and dissemination:**

The study has been approved by the Malawi College of Medicine Research Ethics Committee (COMREC; Ref: P05.19.2680), the University College London Research Ethics Committee (Ref: 8603.002) and the University of Liverpool Research Ethics Committee (Ref: 5439). The results from this study will be actively disseminated through manuscript publications and conference presentations.

**Trial registration number:**

NCT04078997.

**Strengths and limitations of this study**

- The study is supported by more than 20 years of routine pneumococcal surveillance.
- The study design is both pragmatic and robust, offering a methodology that is relevant to diverse settings in the region.
- By engaging community, institutional and government partners, we have optimised its relevance and increased the likelihood of community uptake.
- Limitations to this study include a risk of contamination between clusters (*ie,* children receiving a 2+1 vaccine schedule who reside in or relocate to a cluster implementing the 3+0 vaccine schedule or vice-versa).

## Introduction

*Streptococcus pneumoniae*, the pneumococcus, is commonly carried as a commensal bacterium in the nasopharynx but can cause life-threatening disease. Infections due to the pneumococcus are estimated to be responsible for approximately 300 000 deaths worldwide, with one-third of these occurring among children under 5 years of age and with the greatest burden in low- and middle-income country settings.^1 2^

*S. pneumoniae* has almost 100 serotypes, with nasopharyngeal (NP) carriage as a prerequisite for the development of disease but also a key process for developing natural immunity.^3^ Transmission occurs largely *via* human respiratory air droplets. With serotype-specific differences, carriage duration decreases with age, lasting from 2 weeks in adults up to 4 months among children.^4 5^ Pneumococcal carriage prevalence is age-dependent, peaking among children <5 years. Pneumococcal carriage prevalence reported in sub-Sahara Africa are among the highest described; up to 28% among adults and in excess of 80% in children <5 years old, resulting in high transmission rates.^6–14^

In HICs, routine administration of pneumococcal conjugate vaccines (PCV) through the infant immunisation schedule has contributed to a rapid decline of vaccine serotype invasive pneumococcal disease (VT-IPD) in both vaccinated and unvaccinated populations.^15–23^ PCVs protect the vaccinated individual (direct protection) against pneumococcal disease and carriage. The resulting reduction in carriage also interrupts transmission from the vaccinated individual to the unvaccinated population (indirect protection). The resulting herd immunity effect has been a major contributor to the success of vaccination programmes.^24–26^ The added cost-effectiveness and vaccine impact gained through indirect protection have been key drivers of vaccination policy in these settings.^27 28^ PCV impact on pneumococcal carriage continues to be considered a viable endpoint in vaccine licensure evaluations.^29^

Studies undertaken prior to PCV introduction in The Gambia, Kenya, Mozambique, Malawi and South Africa reported VT carriage prevalence ranging from 28% to nearly 50% among children <5 years old.^30–33^ While PCV introduction in African countries has resulted in substantial direct effects in reducing risk of VT-IPD,^34^ pneumonia and all-cause mortality^35^ among vaccinated children,^10 11 36^ the impact of PCV on VT carriage has been markedly less than that observed in HICs. Although Kenya,^12^ the Gambia,^13^ Mozambique^14^ and South Africa^31^ have reported VT carriage reductions, prevalence remains higher than expected, and serotype replacement (ie, rise in non-VT carriage prevalence associated with decrease in VT carriage) is increasing.^6 37^ As the impact of PCV on carriage is considered an indicator of vaccine impact,^29^ it remains uncertain whether PCV introduction in sub-Sahara Africa will achieve the sustained direct or indirect protection necessary to reduce pneumococcal carriage to levels sufficient to interrupt transmission and disease.^38^

Currently, WHO recommends the implementation of the PCV vaccine using either a 3+0 schedule (three primary doses, most commonly at 6, 10, and 14 weeks of age) or a 2+1 schedule (two primary infancy doses at 6 and 14 weeks of age and one booster at 9 months of age or after the first year of life). The WHO further recommends that the decision on which schedule to use be based on the epidemiology of disease in the local setting.^39^ While both schedules have been shown to be effective in reducing VT disease and VT carriage, there have been no direct comparisons of vaccine impact on carriage in a high burden setting.^40^

A 2017 WHO Technical Expert Consultation on Optimisation of PCV Impact highlighted a substantial research gap in investigating why the impact of PCV vaccines in low-income countries has been less than expected, underlining the need to define an optimal PCV vaccination schedule that will maximise their benefit in such settings.^41^ The PCV Review of Impact Evidence, commissioned to supplement the WHO Expert Consultation, is a systematic review of available evidence on PCV effectiveness and impact on NP carriage, disease and mortality, as well as on PCV immune response. The review included evidence from research studies published between January 2010 and December 2016 recorded in 14 databases. Those attending the 2017 WHO Technical Expert Consultation gave head-to-head studies of 2+1 vs 3+0 vaccine schedules the greatest research priority.^42^ Countries with a high pneumococcal disease and carriage burden, such as Malawi, could implement a 2+1 schedule quickly, with limited logistical or financial demands, providing a booster dose at the time of the first measles vaccine at 9 months of age.

Malawi introduced the 13-valent PCV (PCV13) into the national Expanded Programme of Immunisations (EPI) in November 2011, using a 3+0 schedule (6, 10 and 14 weeks of age), with a three-dose catch-up vaccination campaign among all infants <1 year of age. This introduction has been highly successful, with field studies showing an EPI vaccine coverage exceeding 90%.^43 44^ Similar to other settings, vaccine introduction resulted in a~70% reduction in IPD among PCV-vaccinated children^34^ and an estimated 35% fall in all-cause mortality.^35^ However, high levels of residual VT carriage persist in Malawi among vaccinated children up to 8 years after the introduction of the vaccine.^45^ As presented by Lourenço *et al*^46^ a high force of infection in settings such as Malawi contribute significantly to a 3+0 schedule achieving only a short duration of VT carriage control in infants. Though a 2+1 schedule, as implemented in South Africa, may improve colonisation control, this remains unproven in other African settings such as Malawi.

In this context, the Malawi Ministry of Health (MoH) and the National Immunisations Technical Advisory Group are now seeking evidence of adequate superiority of a 2+1 vaccine schedule to inform a change to Malawi’s current EPI schedule. To this aim, a pragmatic health centre-based evaluation comparing the current 3+0 schedule to a 2+1 schedule will be implemented in Blantyre District, southern Malawi. Two pneumococcal carriage surveys, conducted 18 and 33 months following the implementation of the 2+1 schedule, will have the objective of comparing the effect of the two schedules against carriage reduction among otherwise-healthy children and evaluate their potential to enhance herd immunity.

## Methods and analysis

### Study design

The study is a pragmatic health centre-based randomised evaluation of the direct effect of a 2+1 PCV13 vaccine schedule on pneumococcal carriage in vaccinated infants and the indirect effect on non-vaccine eligible children and high-risk adults.

### Study setting

**Figure 1:**
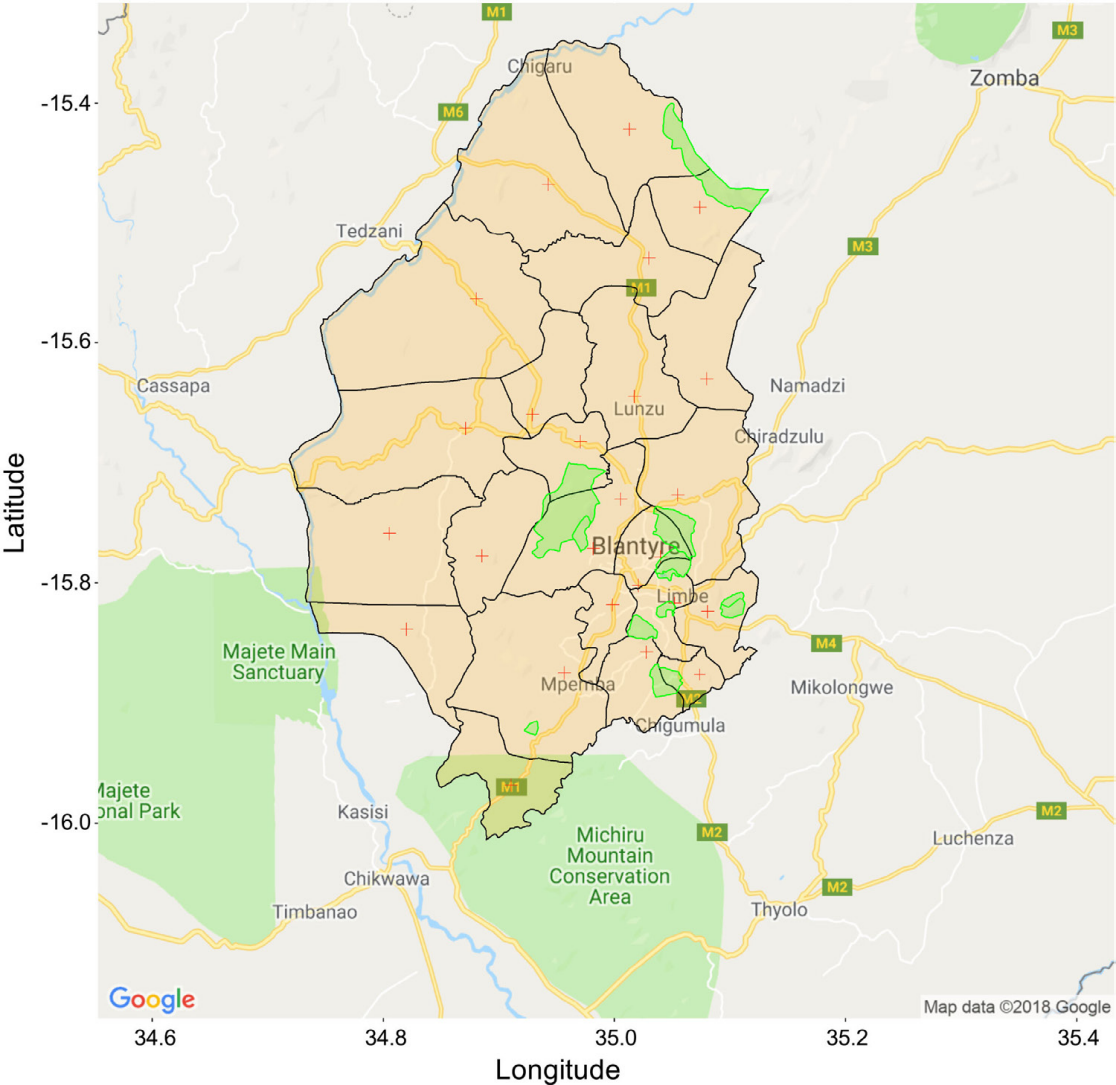
Map of Blantyre district with borders of health centre catchment areas. Red crosses (+) indicate location of health centres offering EPI vaccination. Areas shaded in green are non-inhabited (incl. mountains, industrial zones and other regions administratively declared not for habitation). EPI, Expanded Programme of Immunisations.

The study will be conducted in Blantyre district, southern Malawi. The District is 240 km^2^. Healthcare is delivered through a network of private and government hospitals and 28 government primary health centres, where EPI vaccinations are administered. Queen Elizabeth Central Hospital (QECH) is the government referral hospital providing free medical care to the 1.3 million urban, periurban and rural residents of Blantyre District. Children <5 years account for 16% of the total population.^47^ Health centres cover a fixed geographic area (here referred to as ‘clusters’), and study sampling will be undertaken within these clusters (figure 1).

### Study questions

We will investigate whether a 2+1 PCV13 vaccination schedule (two primary doses at 6 and 14 weeks of age +1 booster dose at 9 months of age), compared with the current 3+0 schedule (three primary doses at 6, 10 and 14 weeks of age with no booster), is superior in reducing VT carriage prevalence among vaccinated children and, consequently, creating a superior herd effect among older children and HIV-infected adults. To assess the effect of a district-wide change from 3+0 to 2+1, this study will address two specific research questions: First, will a 2+1 schedule provide enduring vaccine-induced protection against pneumococcal VT carriage into the second year of life? Second, will the 2+1 vaccine schedule generate stronger herd protection and result in decreased VT carriage in older children and unvaccinated high-risk adults in the general population?

### Primary objective

The primary objective of the study is to evaluate the direct effect of a 2+1 PCV13 vaccination schedule on VT pneumococcal carriage among children aged 15–24 months, 3 years after introducing the 2+1 schedule. This objective will answer the question of whether the 2+1 schedule induces a more enduring direct protection against pneumococcal VT carriage into the second year of life, compared with that observed with a 3+0 schedule.

### Secondary objectives

The secondary objectives of the study are to evaluate, 3 years after introducing the 2+1 schedule, (1) the indirect effect of a 2+1 schedule on VT pneumococcal carriage among children aged 5–10 years (PCV age-ineligible at time of implementing the 2+1 schedule); (2) the indirect effect of a 2+1 vaccination schedule on VT pneumococcal carriage among HIV-infected adults 18–40 years old and on antiretroviral therapy (ART).

To address these questions, the Malawi MoH and Blantyre District Health Office (DHO) will randomly select 10 health centres (among a total 28 in Blantyre District) in which the routine PCV13 schedule will be switched to a 2+1 schedule. An additional 10 health centres will be randomly selected to continue with the current 3+0 schedule but will serve as the direct comparator in evaluating the effectiveness of the 2+1 schedule.

### A pragmatic study design

This switch to a 2+1 schedule is an initiative led by the MoH and will be implemented within the scope of the routine EPI programme, subject to EPI standard procedures for delivery, monitoring and performance assessment. Implementation of all vaccination activities will be implemented, as per routine practices, by MoH EPI vaccinators through the routine EPI programme. The MoH will monitor completeness of dosing following standard reporting practices within the scope of the EPI. Routine study activities throughout the duration of the 3-year study period will include research nurses providing support and guidance to the EPI vaccination teams through weekly site visits. In addition, research enumerators will monitor patient-retained health passports of a representative sample population of vaccinees to confirm they are receiving the proper vaccine schedule (2+1 or 3+0) assigned to the catchment population of their respective health centre.

**Figure 2:**
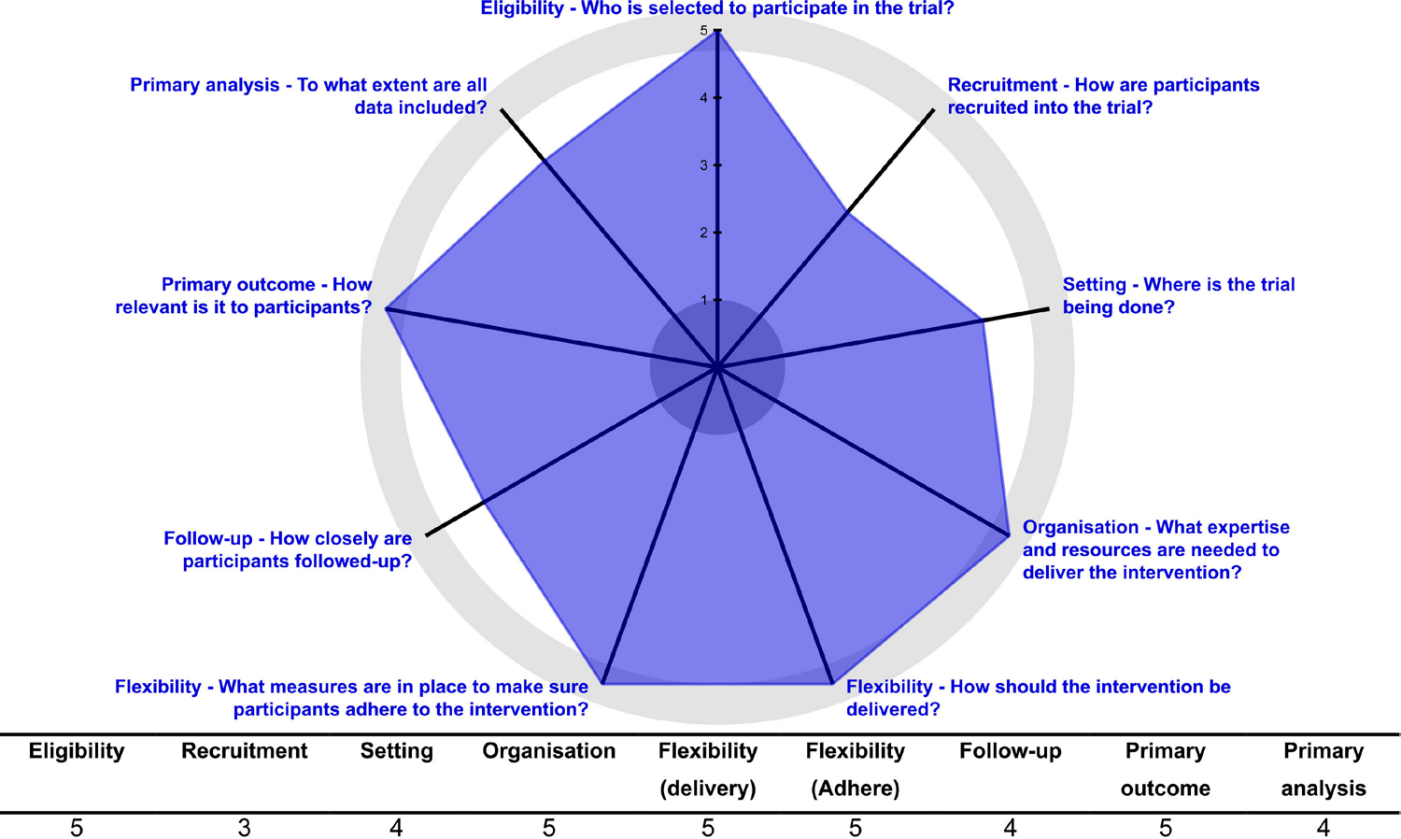
The PRagmatic-Explanatory Continuum Indicator Summary 2 wheel with study-specific scores.

The pragmatic design of this study has been assessed through the tool The PRagmatic-Explanatory Continuum Indicator Summary 2 (PRECIS-2).^48^ This tool was developed to help trialists make design decisions consistent with the intended purpose of their trial. To facilitate domain discussion and consensus, PRECIS-2 assesses the pragmatic design through nine domains—eligibility criteria, recruitment, setting, organisation, flexibility (delivery), flexibility (adherence), follow-up, primary outcome and primary analysis—scored from 1 (very explanatory) to 5 (very pragmatic). The report from this tool is in the format of a wheel (figure 2). Results (mean score: 4.4 and range: 3–5) supports that this is largely a pragmatic randomised study undertaken in the ‘real world’ and with usual care and is intended to help support a decision on whether to deliver an intervention.

### 3+1 schedule during early period of initiation

To ensure the 2+1 schedule is implemented efficiently, and to limit the risk of confusion during the transition to the 2+1 schedule (including not deferring PCV13 vaccination to a later visit), a 3+1 schedule will be implemented during the first several months at health centres implementing the 2+1 schedule. At these health centres, children who have received either their 10 weeks or 14 weeks PCV13 dose before 2+1 implementation will receive a dose at their 14 weeks (third dose PCV13) and their 40 weeks (fourth dose PCV13). Approximately 6 months after 2+1 implementation, all first-contact vaccine visits (post 2+1 implementation) will be for the scheduled visit at 6 weeks of age and the 3+1 schedule will no longer be required.

### Carriage surveys

**Figure 3:**
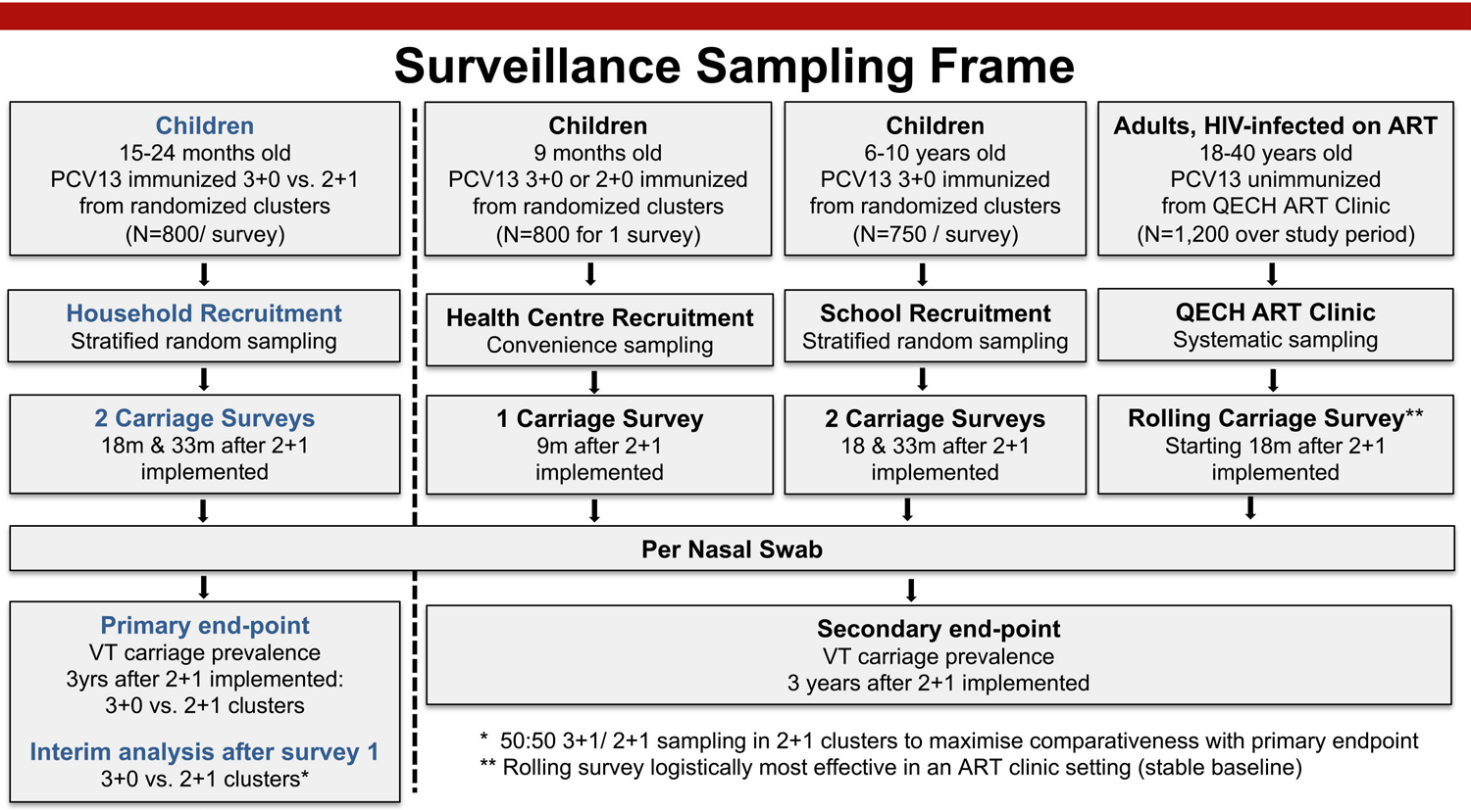
Carriage surveillance sampling frame black dashed vertical line separates primary (left of dashed line) from secondary study objectives. ART, antiretroviral; M, months; PCV, pneumococcal conjugate; QECH, Queen Elizabeth Central Hospital.

The study will include two cross-sectional carriage surveys, implemented 18 and 33 months after the switch to 2+1. Carriage surveys will be conducted using a well-established methodology implemented extensively in the setting,^45 46^ allowing comparability of study results to previous carriage surveys conducted in the area. The sampling frame is illustrated in figure 3.

Sampling will include:

1. Children 15–24 months of age, PCV13-vaccinated (confirmed by reviewing patient-retained health passports) with either the 2+1 or the 3+0 schedule. Evaluation within this age group will allow us to evaluate for our primary objective, evaluating the direct effect of vaccination on carriage. Within each of the catchment areas of the 20 participating health centres, we will conduct a random walk method to systematically identify households with eligible children. A ‘egg and yolk’ strategy will be applied, defining two geographic sampling perimeters around each health centre. Sampling will prioritise those living within the geographic perimeter closest to the health centre. In the event that recruitment targets are not met, sampling will move into the second geographic perimeter. This strategy will maximise the likelihood of recruiting children who have received their vaccines at the selected health centre, minimising recruitment from buffer zonal borders and therefore minimising risk of contamination (ie, recruitment of children receiving a PCV13 vaccine schedule different than the schedule assigned to the child’s local health centre).
2. Children 5–10 years of age, PCV13-vaccinated with the 3+0 schedule. These children, having received PCV13 at infancy, will be recruited from government schools. Evaluation within this age group will allow us to evaluate for a secondary objective, evaluating the indirect effect of a 2+1 schedule on VT pneumococcal carriage among children aged 5–10 years, the majority of whom received PCV13 infancy EPI vaccination using a 3+0 schedule. Six schools will be selected, three located centrally in each of the 3+0 clusters and the 2+1 clusters. Children will be chosen at random from school registers. This component will allow the evaluation of indirect effects of the 2+1 compared with 3+0 schedule among children with waning vaccine-induced immunity.
3. Adults 18–40 years of age, HIV-infected on ART and PCV13-unvaccinated. Evaluation within group will allow us to achieve the secondary objective of evaluating the indirect effect of a 2+1 schedule on VT pneumococcal carriage among HIV-infected adults (18–40 years) on ART, a population at high risk of pneumococcal disease. Participants will be recruited from the QECH, Lighthouse ART Clinic in Blantyre.

### Safety

To assess the differential effects of 3 vs 2 primary vaccine doses in the first year of life, an additional carriage survey will be implemented at health centres approximately 9 months after 2+1 implementation. A convenience sample of children aged 9 months will be recruited at health centres providing either the 2+1 or 3+0 schedule. This will allow an evaluation of carriage prevalence among children receiving 3 (according to the 3+0 schedule) vs 2 (according to the 2+1 schedule and presenting for their booster dose) primary doses. Additionally, this additional carriage survey will contribute to evaluating the coverage of the 2+1 vaccination schedule and the proportion of children receiving all three PCV13 doses at health centres providing the 2+1 schedule.

### Inclusion and exclusion criteria

Inclusion criteria for all individuals recruited includes permanent resident in Blantyre District. Among children 15–24 months of age (assigned to either 2+1 or 3+0 schedule), inclusion criteria additionally include: aged between 15 and 24 months, parent/legal guardian providing written informed consent, evidence (recorded in health passport) of having received a full schedule of PCV13. Among children 5–10 years of age, inclusion criteria include: aged between 5–10 years, parent/legal guardian providing written informed consent, providing written informed assent (if the child is aged ≥8), and either verbal or documented evidence of having received PCV13. Among adults, inclusion criteria include: aged 18–40 years, providing written informed consent, and being HIV-infected and receiving ART.

Among children 9 months of age, recruited as part of the safety component, inclusion criteria include: aged 9 months, parent/legal guardian providing written informed consent, and evidence (recorded in health passport) of having received either a full 3+0 PCV13 vaccine schedule (health centres implementing 3+0) or both primary doses of PCV13 at approximately 6 and 14 weeks of age (health centres implementing 2+1).

Exclusion criteria for all screened individuals include receiving TB treatment at time of screening, hospitalisation for pneumonia within 14 days prior to study screening and terminal illness. Exclusion criteria for all children include parental/legal guardian not providing consent, not providing assent (for children aged ≥8 years), having received antibiotic treatment within 14 days prior to study screening. Exclusion criteria for adults include not providing written informed consent or prior vaccination with a pneumococcal vaccine.

### Intervention

The intervention will consist of two carriage surveys conducted 18 and 33 months after the 10 randomised health centres switch from a 3+0 to a 2+1 PCV13 schedule. In both surveys, a single NP swab will be collected from each participant. Following previously described WHO-recommended procedures,^45 49^ these will be collected and taken to the Malawi-Liverpool-Wellcome Trust Clinical Research Programme laboratory in Blantyre for the isolation and characterisation of *S. pneumoniae*. VT pneumococcal carriage will be serotyped by latex agglutination. Samples from participants with confirmed pneumococcal carriage will be sent to the UK for assessment of multiple serotype carriage (genomic microarray) and for whole genome sequencing.

### Expected outcomes

The primary endpoint of this study will be the difference in VT pneumococcal carriage prevalence among children aged 15–24 months, comparing those vaccinated with PCV13 in either a 2+1 or 3+0 schedule, 3 years after implementing the switch to a 2+1 schedule. Additionally, the study will evaluate four secondary outcomes: (1) difference in VT carriage prevalence among children 5–10 years old, 18 months and 33 months after 2+1 implementation, (2) VT carriage prevalence among HIV-infected adults aged 18–40 years and receiving ART at the time of sampling, (3) VT carriage prevalence among infants aged 9 months, who will have received three primary doses (3+0 health centres) or two primary doses (2+1 health centres) prior to the booster dose, 9 months after the implementation of the 2+1 schedule and (4) prevalence of multiple serotype carriage, 18 and 33 months after 2+1 implementation

### Patient and public involvement statement

Prior to development of the protocol, key stakeholders were informed of the study, including the study sites (ie, selected health centres) and their surrounding communities (ie, catchment areas), the DHO, MoH and the Ministry of Education. We actively sought and incorporated input from these stakeholders into the study objectives and overall design. Community sensitisation will be further strengthened through a community advisory board.

### Statistical methods

#### Study power and sample size calculation

The primary endpoint is powered to detect an effect size of 50% reduction in VT carriage, expecting a 14% and 7% VT carriage prevalence among vaccinated children 15–24 months old in the 3+0 and 2+1 arms, respectively. Sample sizes were calculated based on a power of 80% and a statistical significance of 0.05. The calculations accounted for household similarities using an intraclass correlation (ICC) of 0.005 (based on previous experience in this setting) and adjusted for a design effect (dependent on both ICC and cluster size.) of 1.21. Minimum sample sizes needed to achieve the necessary power under these assumptions are shown in table 1.

**Table 1:** Sample size estimations

N per cluster	ICC	p1	p2	Power	Alpha	DE	N per arm	Clusters, per arm	Clusters, total	N total
40	0.005	0.14	0.07	0.8	0.05	1.20	358	9	18	720
40	0.005	0.14	0.07	0.8	0.05	1.21	400	10	20	800
45	0.005	0.14	0.07	0.8	0.05	1.22	366	9	18	810
50	0.005	0.14	0.07	0.8	0.05	1.25	373	8	16	800
55	0.005	0.14	0.07	0.8	0.05	1.27	381	7	14	770
60	0.005	0.14	0.07	0.8	0.05	1.30	388	7	14	840
40	0.01	0.14	0.07	0.8	0.05	1.39	417	11	22	880
45	0.01	0.14	0.07	0.8	0.05	1.44	432	10	20	900
50	0.01	0.14	0.07	0.8	0.05	1.49	447	9	18	900
55	0.01	0.14	0.07	0.8	0.05	1.54	462	9	18	990
60	0.01	0.14	0.07	0.8	0.05	1.59	477	8	16	960
40	0.015	0.14	0.07	0.8	0.05	1.59	475	12	24	960

DE, design effect; ICC, intraclass correlation.

To assess the primary and secondary endpoints (1) One child 15–24 months of age (2+1 or 3+0) will be recruited from each of 40 households randomly selected within each of the 20 clusters. This is a total 800 vaccinated children per survey (1600 total for two surveys). (2) A total of 125 children will be recruited from each of the six schools per survey. This is a total 750 children per survey (1500 total). (3) A total of 1200 HIV-infected adults will be recruited from the QECH ART Clinic over the course of the 3-year study period. (4) A total of 800 children 9 months of age will be recruited from the vaccination centres 9 months after schedule change (800 total).

#### Data collection, management and anonymisation procedures

Demographic data and relevant medical history will be collected using password-protected electronic data capturing. Each participant will be assigned a unique participant identification number (PID) at recruitment. This PID will be used in all datasheets and files, and will be linked to the laboratory data, hence, only anonymised data will be used for the analysis. Fully anonymised data will be uploaded daily to a secured on-site server, which is backed up daily to both local and off-site facilities. A logbook containing identifiable information (including name) will be kept separate in a secured location by an authorised member of the study team and will only be accessed by authorised study members. This will allow the study team to recover any missing epidemiological information at a later date (eg, missing vaccination dates) and to facilitate any participants who choose to withdraw consent at any time.

### Statistical analyses

Continuous variables will be summarised by means and SD, or medians and IQRs if the distribution exhibits skew. Categorical variables will be summarised by frequency distributions. Direct effects of the PCV13 schedule change on VT carriage will be ascertained by comparing carriage prevalence in children aged 15–24 months residing in the recruitment clusters of health centres randomised to the 2+1 schedule and those in recruitment clusters of health centres randomised to the 3+0 schedule. Indirect effects will be evidenced by comparing children in the recruitment clusters of health centres randomised to the 3+0 schedule among older children (5–10 years) and HIV +adults. Additionally, data obtained in this study will be compared with those obtained from previous carriage surveys to ascertain any changes in VT carriage prevalence before and after the PVC13 schedule change. Statistical tests will be selected depending on the distribution patterns of the data. Potential confounders and sources of interaction (including age, gender and health centre) will be identified by testing the association between variables and VT carriage and included in the multivariable models when p<0.1. Sensitivity analyses will include assessing impact of (1) receiving only one, only two or all three doses PCV; (2) having document-confirmed PCV vaccination or (3) schedule adherence to within 2 weeks of each scheduled dose on VT prevalence and on the VT distribution.

## Ethics and dissemination

### Ethics approvals

The study has been approved by the Malawi College of Medicine Research Ethics Committee (COMREC; Ref: P05.19.2680), the University College London Research Ethics Committee (Ref: 8603.002) and the University of Liverpool Research Ethics Committee (Ref: 5439).

### Data monitoring external advisory group

The implementation of the study protocol will be reviewed and monitored by an External Advisory Group, providing oversight of the study activities and advise the study team at the time of the interim and final analyses. The group will include experts from the University of Malawi, College of Medicine, the London School of Hygiene & Tropical Medicine and the Medical Research Council.

### Interim analysis and changes to public health policy

On completion of the first carriage survey, an interim analysis will be implemented using data obtained on the VT carriage prevalence among vaccinated children 15–24 months of age. These results will be used to assess possible adaptation of the second community carriage survey. Three possible scenarios are considered: (1) If convincing evidence of major change in carriage prevalence is demonstrated, immediate action will be discussed with the MoH to move to change the schedule and adapt year-3 sampling; (2) If no major change in carriage prevalence is identified, the study will continue as planned; (3) If carriage prevalence has fallen by more than 30% but does not meet the primary threshold of 50%, the second carriage survey will be brought forward by 6 months. Decisions will be reached in consensus between investigators, the Expert Advisory group, and the MoH.

### Informed consent process

The study will only recruit children whose parents/legal guardians have the capacity to provide informed consent or, in the case of adults, are capable of giving consent. Children who are minors but ≥8 years of age will be required to provide informed assent, in addition to parents/legal guardians providing informed consent. Participants will receive both verbal and written information about the study and will be given the opportunity to ask questions and express their doubts and concerns before accepting to take part. They will also be given time to reflect before they come to a decision. An informed consent and/or assent form will be signed and dated by the participant and a member of the research team. The participant will keep a copy of the document, and a second one will be kept in the study file with the Principal Investigator based in Blantyre, Malawi. Participants will be informed of their right to withdraw consent at any point until study ends without the need to provide a reason and without penalty.

## Dissemination policy and plans

Study results will be shared with local stakeholders and published in peer-reviewed journals. Partial results and interim analyses will be shared with the Malawi MoH and other relevant policymakers and decision-making stakeholders. Partial and final findings will be presented at relevant international conferences and meetings. Copies of all published materials and reports will be shared with the research ethics committees and collaborators. We will return to the community partners and work with the community advisory board to report and further disseminate our results into those communities where we worked.

## Supplementary Material

Reviewer comments

Author's manuscript
