# Supplementary material for: A pragmatic health centre-based evaluation comparing the effectiveness of a PCV13 schedule change from 3+0 to 2+1 in a high pneumococcal carriage and disease burden setting in Malawi: a study protocol
Source: BMJ Open. 2021 Jun 17;11(6):e050312. doi: 10.1136/bmjopen-2021-050312 (PMC8212416; doi:10.1136/bmjopen-2021-050312)
Supplement: Author's manuscript [file bmjopen-2021-050312.draft_revisions.pdf]

BMJ Open is committed to open peer review. As part of this commitment we make the peer review history of every article we publish publicly available.

When an article is published we post the peer reviewers' comments and the authors' responses online. We also post the versions of the paper that were used during peer review. These are the versions that the peer review comments apply to.

The versions of the paper that follow are the versions that were submitted during the peer review process. They are not the versions of record or the final published versions. They should not be cited or distributed as the published version of this manuscript.

BMJ Open is an open access journal and the full, final, typeset and author-corrected version of record of the manuscript is available on our site with no access controls, subscription charges or pay-per-view fees (http://bmjopen.bmj.com).

If you have any questions on BMJ Open's open peer review process please email info.bmjopen@bmj.com

BMJ Open

A pragmatic health centre-based evaluation comparing the effectiveness of a PCV13 schedule change from 3+0 to 2+1 in a high pneumococcal carriage and disease burden setting in Malawi: a study protocol

Journal:	*BMJ Open*
Manuscript ID	bmjopen-2021-050312
Article Type:	Protocol
Date Submitted by the Author:	17-Feb-2021

Complete List of Authors:	Swarthout, Todd; Malawi-Liverpool-Wellcome Trust Clinical Research Programme; University College London<br>Ibarz-Pavon, Ana; Malawi-Liverpool-Wellcome Trust Clinical Research Programme; University of Liverpool<br>Kawalazira, Gift; Ministry of Health<br>Sinjani, George; Malawi-Liverpool-Wellcome Trust Clinical Research Programme<br>Chirombo, James; Malawi-Liverpool-Wellcome Trust Clinical Research Programme<br>Gori, Andrea; University College London<br>Chalusa, Peter; Malawi-Liverpool-Wellcome Trust Clinical Research Programme<br>Bonomali, Farouck; Malawi-Liverpool-Wellcome Trust Clinical Research Programme<br>Nyirenda, Roseline; Malawi-Liverpool-Wellcome Trust Clinical Research Programme<br>Bulla, Edwin; Malawi-Liverpool-Wellcome Trust Clinical Research Programme<br>Brown, Comfort; Malawi-Liverpool-Wellcome Trust Clinical Research Programme<br>Msefula, Jacquline; Malawi-Liverpool-Wellcome Trust Clinical Research Programme<br>Banda, Marjory; Ministry of Education<br>Kachala, Jean; Ministry of Health<br>Mwansambo, Charles; Ministry of Health<br>Henrion, Marc; Malawi Liverpool Wellcome Trust Clinical Research Programme; Liverpool School of Tropical Medicine, Department of Clinical Sciences<br>Gordon, Stephen; University of Malawi College of Medicine, Malawi-Liverpool-Wellcome Trust Clinical Research Programme; Liverpool School of Tropical Medicine Department of Clinical Sciences, Clinical Sciences<br>French, Neil; University of Liverpool Faculty of Health and Life Sciences, Centre for Global Vaccine Research, Institute of Infection and Global Health; University of Malawi College of Medicine, Malawi-Liverpool-Wellcome Trust Clinical Research Programme<br>Heyderman, Robert; University College London, Division of Infection and Immunity; University of Malawi College of Medicine, Malawi Liverpool Wellcome Trust Clinical Research Programme

Keywords:	EPIDEMIOLOGY, Epidemiology < INFECTIOUS DISEASES, Public health < INFECTIOUS DISEASES, Paediatric infectious disease & immunisation < PAEDIATRICS, PUBLIC HEALTH

I, the Submitting Author has the right to grant and does grant on behalf of all authors of the Work (as defined in the below author licence), an exclusive licence and/or a non-exclusive licence for contributions from authors who are: i) UK Crown employees; ii) where BMJ has agreed a CC-BY licence shall apply, and/or iii) in accordance with the terms applicable for US Federal Government officers or employees acting as part of their official duties; on a worldwide, perpetual, irrevocable, royalty-free basis to BMJ Publishing Group Ltd ("BMJ") its licensees and where the relevant Journal is co-owned by BMJ to the co-owners of the Journal, to publish the Work in this journal and any other BMJ products and to exploit all rights, as set out in our licence.

The Submitting Author accepts and understands that any supply made under these terms is made by BMJ to the Submitting Author unless you are acting as an employee on behalf of your employer or a postgraduate student of an affiliated institution which is paying any applicable article publishing charge ("APC") for Open Access articles. Where the Submitting Author wishes to make the Work available on an Open Access basis (and intends to pay the relevant APC), the terms of reuse of such Open Access shall be governed by a Creative Commons licence – details of these licences and which Creative Commons licence will apply to this Work are set out in our licence referred to above.

Other than as permitted in any relevant BMJ Author's Self Archiving Policies, I confirm this Work has not been accepted for publication elsewhere, is not being considered for publication elsewhere and does not duplicate material already published. I confirm all authors consent to publication of this Work and authorise the granting of this licence.

For peer review only

A pragmatic health centre-based evaluation comparing the effectiveness of a PCV13 schedule change from 3+0 to 2+1 in a high pneumococcal carriage and disease burden setting in Malawi: a study protocol

Todd D. Swarthout[1,2,¥,#], Ana Ibarz-Pavon[1,3¥], Gift Kawalazira[4], George Sinjani[1], James Chirombo[1], Andrea Gori[2], Peter Chalusa[1], Farouck Bonomali[1], Roseline Nyirenda[1], Edwin Bulla[1], Comfort Brown[1], Jacquline Msefula[1], Marjory Banda[5], Jean Kachala[4], Charles Mwansambo[4], Marc Y.R. Henrion[1,3], Stephen B Gordon[1], Neil French[1,4*], Robert S. Heyderman[1,2*]

Author Affiliations

[1]Malawi-Liverpool-Wellcome Trust Clinical Research Programme, Blantyre, Malawi

[2]NIHR Global Health Research Unit on Mucosal Pathogens, Division of Infection and Immunity, University College London, London, United Kingdom

[3]Department of Clinical Sciences, Liverpool School of Tropical Medicine, Liverpool, United Kingdom

[4]Centre for Global Vaccine Research, Institute of Infection, Veterinary, and Ecological Science, University of Liverpool, Liverpool, United Kingdom

[4]Ministry of Health, Lilongwe, Malawi

[5]Ministry of Education, Blantyre, Malawi

¥Todd D. Swarthout and Ana Ibarz-Pavon have contributed equally to this manuscript

* Neil French and Robert Heyderman have contributed equally to this manuscript

#Corresponding author: Todd Swarthout, Malawi-Liverpool-Wellcome Trust Clinical Research Programme, P.O. Box 30096, Blantyre, Malawi; Email: t.swarthout@ucl.ac.uk; Phone: +265 (0)1 874 628

For peer review only

Abstract

Introduction. *Streptococcus pneumoniae* (the pneumococcus) is commonly carried as a commensal bacterium in the nasopharynx but can cause life-threatening disease. Transmission occurs by human respiratory droplets and interruption of this process provides herd immunity. A 2017 WHO Consultation on Optimization of PCV Impact highlighted a substantial research gap in investigating why the impact of PCV vaccines

in low-income countries has been lower than expected. Malawi introduced the 13-valent PCV (PCV13) into the national Expanded Program of Immunizations (EPI) in 2011, using a 3+0 (3 primary + 0 booster doses) schedule. With evidence of greater impact of a 2+1 (2 primary + 1 booster dose) schedule in other settings, including South Africa, Malawi's National Immunizations Technical Advisory Group is seeking evidence of adequate superiority of a 2+1 schedule to inform vaccine policy.

Methods. A pragmatic health centre-based evaluation comparing impact of a PCV13 schedule change from 3+0 to 2+1 in Blantyre District, Malawi. Twenty government health centres will be randomly selected, with ten implementing a 2+1 and ten to continue with the 3+0 schedule. Health centres implementing 3+0 will serve as the direct comparator in evaluating 2+1 providing superior direct and indirect protection against pneumococcal carriage. Pneumococcal carriage surveys will evaluate carriage prevalence among children 15-24 months, randomized at household level, and schoolgoers 5-10 years of age, randomly selected from school registers. Carriage surveys will be conducted 18 and 33 months following 2+1 implementation.

Analysis. The primary endpoint is powered to detect an effect size of 50% reduction in VT carriage among vaccinated children 15-24 months old, expecting a 14% and 7% VT carriage prevalence in the 3+0 and 2+1 arms, respectively.

Ethics and dissemination. The study has been approved by the Malawi College of Medicine Research Ethics Committee (COMREC; Ref: P05.19.2680), the University College London Research Ethics Committee (Ref: 8603.002) and the University of Liverpool Research Ethics Committee (Ref: 5439). The results from this study will be actively disseminated through manuscript publications and conference presentations.

Trial Registration number NCT04078997.

Article Summary

Strengths and limitations of this study

- The study is supported by more than 20 years of routine pneumococcal surveillance.
- The study design is both pragmatic and robust, offering a methodology that is relevant to diverse settings in the region.

For peer review only

For peer review only - http://bmjopen.bmj.com/site/about/guidelines.xhtml

- By engaging community, institutional and government partners, we have optimised its relevance and increased the likelihood of community uptake.
- Limitations to this study include a risk of contamination between clusters (*i.e.* children receiving a 2+1 vaccine schedule who reside in or relocate to a cluster implementing the 3+0 vaccine schedule, or *vice-versa*).

Key Words: *Streptococcus pneumoniae*; immunity, herd; 13-valent pneumococcal vaccine; Africa; Malawi; pneumococcal vaccines; immunization; schedule

For peer review only

For peer review only - http://bmjopen.bmj.com/site/about/guidelines.xhtml

Introduction

Streptococcus pneumoniae, the pneumococcus, is commonly carried as a commensal bacterium in the nasopharynx but can cause life-threatening disease. Infections due to the pneumococcus are estimated to be responsible for approximately 300,000 deaths worldwide, with one-third of these occurring among children under five years of age and with the greatest burden in low- and middle-income country settings (1,2).

S. pneumoniae has almost 100 serotypes, with nasopharyngeal (NP) carriage as a pre-requisite for the development of disease but also a key process for developing natural immunity (3). Transmission occurs largely *via* human respiratory air droplets. With serotype-specific differences, carriage duration decreases with age, lasting from 2 weeks in adults up to 4 months among children (4,5). Pneumococcal carriage prevalence is age-dependent, peaking among children <5 years. Pneumococcal carriage prevalence reported in sub-Sahara Africa are among the highest described; up to 28% among adults and in excess of 80% in children <5 years old, resulting in high transmission rates (6–14).

In HICs, routine administration of pneumococcal conjugate vaccines (PCV) through the infant immunization schedule has contributed to a rapid decline of vaccine serotype invasive pneumococcal disease (VT-IPD) in both vaccinated and unvaccinated populations (15–23). PCVs protect the vaccinated individual (direct protection) against pneumococcal disease and carriage. The resulting reduction in carriage also interrupts transmission from the vaccinated individual to the unvaccinated population (indirect protection). The resulting herd immunity effect has been a major contributor to the success of vaccination programs (24–26). The added cost-effectiveness and vaccine impact gained through indirect protection have been key drivers of vaccination policy in these settings (27,28). PCV impact on pneumococcal carriage continues to be considered a viable endpoint in vaccine licensure evaluations (29).

Studies undertaken prior to PCV introduction in The Gambia, Kenya, Mozambique, Malawi and South Africa reported VT carriage prevalence ranging from 28% to nearly 50% among children <5 years old (30–33). While PCV introduction in African countries

For peer review only - http://bmjopen.bmj.com/site/about/guidelines.xhtml

has resulted in substantial direct effects in reducing risk of VT-IPD (34), pneumonia and all-cause mortality (35) among vaccinated children (10,11,36), the impact of PCV on VT carriage has been markedly less than that observed in HICs. Although Kenya (12), the Gambia (13), Mozambique (14), and South Africa (31) have reported VT carriage reductions, prevalence remains higher than expected, and serotype replacement (i.e. rise in non-VT (NVT) carriage prevalence associated with decrease in VT carriage) is increasing (6,37). As the impact of PCV on carriage is considered an indicator of vaccine impact (29), it remains uncertain whether PCV introduction in sub-Sahara Africa will achieve the sustained direct or indirect protection necessary to reduce pneumococcal carriage to levels sufficient to interrupt transmission and disease (38).

Currently, the World Health Organization (WHO) recommends the implementation of the PCV vaccine using either a or a 3+0 schedule (three primary doses, most commonly at 6, 10, and 14 weeks of age) or a 2+1 schedule (two primary infancy doses at 6 and 14 weeks of age and one booster at 9 months of age or after the 1st year of life). The WHO further recommends that the decision on which schedule to use be based on the epidemiology of disease in the local setting (39). While both schedules have been shown to be effective in reducing VT disease and VT carriage, there have been no direct comparisons of vaccine impact on carriage in a high burden setting (40).

A 2017 WHO Technical Expert Consultation on Optimization of PCV Impact highlighted a substantial research gap in investigating why the impact of PCV vaccines in low-income countries has been less than expected, underlining the need to define an optimal PCV vaccination schedule that will maximize their benefit in such settings (41). The PCV Review of Impact Evidence (PRIME), commissioned to supplement the WHO Expert Consultation, is a systematic review of available evidence on PCV effectiveness and impact on nasopharyngeal carriage, disease and mortality, as well as on PCV immune response. The review included evidence from research studies published between January 2010-December 2016 recorded in 14 databases. Those attending the 2017 WHO Technical Expert Consultation gave head-to-head studies of 2+1 vs 3+0 vaccine schedules the greatest research priority (42). Countries with a high pneumococcal disease and carriage burden, such as Malawi, could implement a

For peer review only - http://bmjopen.bmj.com/site/about/guidelines.xhtml

2+1 schedule quickly, with limited logistical or financial demands, providing a booster dose at the time of the first measles vaccine at 9 months of age.

Malawi introduced the 13-valent PCV (PCV13) into the national Expanded Program of Immunizations (EPI) in November 2011, using a 3+0 schedule (6, 10 and 14 weeks of age), with a three-dose catch-up vaccination campaign among all infants <1 year of age. This introduction has been highly successful, with field studies showing an EPI vaccine coverage exceeding 90% (43,44). Similar to other settings, vaccine introduction resulted in a ~70% reduction in IPD among PCV-vaccinated children (34) and an estimated 35% fall in all-cause mortality (35). However, high levels of residual VT carriage persist in Malawi among vaccinated children up to eight years after the introduction of the vaccine (45). As presented by Lourenço *et. al*. (46), a high force of infection in settings such as Malawi contribute significantly to a 3+0 schedule achieving only a short duration of VT carriage control in infants. Though a 2+1 schedule, as implemented in South Africa, may improve colonisation control, this remains unproven in other African settings such as Malawi.

In this context, the Malawi Ministry of Health (MoH) and the National Immunizations Technical Advisory Group (NITAG) are now seeking evidence of adequate superiority of a 2+1 vaccine schedule to inform a change to Malawi's current EPI schedule. To this aim, a pragmatic health centre-based evaluation comparing the current 3+0 schedule to a 2+1 schedule will be implemented in Blantyre District, southern Malawi. Two pneumococcal carriage surveys, conducted 18 and 33 months following the implementation of the 2+1 schedule, will compare the effect of the two schedules against carriage reduction among otherwise-healthy children and evaluate their potential to enhance herd immunity.

For peer review only - http://bmjopen.bmj.com/site/about/guidelines.xhtml

Methods and analysis

Study design: The study is a pragmatic health centre-based randomized evaluation of the direct effect of a 2+1 PCV13 vaccine schedule on pneumococcal carriage in vaccinated infants and the indirect effect on non-vaccine eligible children and high-risk adults.

Study setting. The study will be conducted in Blantyre District, southern Malawi. The District is 240 Km^2. Healthcare is delivered through a network of private and government hospitals and 28 government primary health centres, where EPI vaccinations are administered. Queen Elizabeth Central Hospital (QECH) is the government referral hospital providing free medical care to the 1·3 million urban, peri-urban and rural residents of Blantyre District. Children <5 years account for 16% of the total population (47). Health centres cover a fixed geographic area (here referred to as 'clusters'), and study sampling will be undertaken within these clusters (Figure 1).

Study questions. We will investigate whether a 2+1 PCV13 vaccination schedule (2 primary doses at 6 and 14 weeks of age + 1 booster dose at 9 months of age), compared to the current 3+0 schedule (3 primary doses at 6, 10 and 14 weeks of age with no booster), is superior in reducing VT carriage prevalence among vaccinated children and, consequently, creating a superior herd effect among older children and HIV-infected adults. To assess the effect of a district-wide change from 3+0 to 2+1, this study will address two specific research questions: Firstly, will a 2+1 schedule provide enduring vaccine-induced protection against pneumococcal VT carriage into the second year of life? Secondly, will the 2+1 vaccine schedule generate stronger herd protection and result in decreased VT carriage in older children and unvaccinated high-risk adults in the general population?

Primary objective. The primary objective of the study is to evaluate the direct effect of a 2+1 PCV13 vaccination schedule on VT pneumococcal carriage among children aged 15-24 months, three years after introducing the 2+1 schedule. This objective will answer the question of whether the 2+1 schedule induces a more enduring direct

For peer review only - http://bmjopen.bmj.com/site/about/guidelines.xhtml

protection against pneumococcal VT carriage into the second year of life, compared to that observed with a 3+0 schedule.

Secondary objectives. The secondary objectives of the study are to evaluate, three years after introducing the 2+1 schedule, i) the indirect effect of a 2+1 schedule on VT pneumococcal carriage among children aged 5-10 years (PCV age-ineligible at time of implementing the 2+1 schedule; ii) the indirect effect of a 2+1 vaccination schedule on VT pneumococcal carriage among HIV-infected adults 18-40 years old and on antiretroviral therapy (ART).

To address these questions, the Malawi MoH and Blantyre District Health Office (DHO) will randomly select 10 health centres (among a total 28 in Blantyre District) in which the routine PCV13 schedule will be switched to a 2+1 schedule. An additional 10 health centres will be randomly selected to continue with the current 3+0 schedule but will serve as the direct comparator in evaluating the effectiveness of the 2+1 schedule.

A pragmatic study design. This switch to a 2+1 schedule is an initiative led by the MoH and will be implemented within the scope of the routine EPI programme, subject to EPI standard procedures for delivery, monitoring, and performance assessment. Implementation of all vaccination activities will be implemented, as per routine practices, by MoH EPI vaccinators through the routine EPI programme. The MoH will monitor completeness of dosing following standard reporting practices within the scope of the EPI. Routine study activities throughout the duration of the three-year study period will include research nurses providing support and guidance to the EPI vaccination teams through weekly site visits. In addition, research enumerators will monitor patient-retained health passports of a representative sample population of vaccinees to confirm they are receiving the proper vaccine schedule (2+1 or 3+0) assigned to the catchment population of their respective health centre.

The pragmatic design of this study has been assessed through the PRECIS-2 tool (The PRagmatic-Explanatory Continuum Indicator Summary 2) (48). This tool was developed to help trialists make design decisions consistent with the intended purpose of their trial. To facilitate domain discussion and consensus, PRECIS-2

For peer review only - http://bmjopen.bmj.com/site/about/guidelines.xhtml

assesses the pragmatic design through nine domains - eligibility criteria, recruitment, setting, organisation, flexibility (delivery), flexibility (adherence), follow-up, primary outcome, and primary analysis - scored from 1 (very explanatory) to 5 (very pragmatic). The report from this tool is in the format of a wheel (Figure 2). Results (mean score: 4.4 and range: 3-5) supports that this is largely a pragmatic randomised study undertaken in the "real world" and with usual care and is intended to help support a decision on whether to deliver an intervention.

3+1 schedule during early period of initiation. To ensure the 2+1 schedule is implemented efficiently, and to limit the risk of confusion during the transition to the 2+1 schedule (including not deferring PCV13 vaccination to a later visit), a 3+1 schedule will be implemented during the first several months at health centres implementing the 2+1 schedule. At these health centres, children who have received either their 10-week or 14-week PCV13 dose before 2+1 implementation will receive a dose at their 14-week (3rd dose PCV13) and their 40-week (4th dose PCV13). Approximately 6 months after 2+1 implementation, all first-contact vaccine visits (post 2+1 implementation) will be for the scheduled visit at 6-weeks of age and the 3+1 schedule will no longer be required.

Carriage surveys. The study will include two cross-sectional carriage surveys, implemented 18 and 33 months after the switch to 2+1. Carriage surveys will be conducted using a well-established methodology implemented extensively in the setting (45,46), allowing comparability of study results to previous carriage surveys conducted in the area. The sampling frame is illustrated in Figure 3.

Sampling will include:

i) children 15-24 months of age, PCV13-vaccinated (confirmed by reviewing patient-retained health passports) with either the 2+1 or the 3+0 schedule. Evaluation within this age group will allow us to evaluate for our primary objective, evaluating the direct effect of vaccination on carriage. Within each of the catchment areas of the 20 participating health centres, we will conduct a random walk method to systematically identify households with eligible children. A 'egg and yolk' strategy will be applied, defining two geographic sampling perimeters around each health centre. Sampling will prioritize those living within the geographic perimeter closest to the health centre. In

For peer review only - http://bmjopen.bmj.com/site/about/guidelines.xhtml

the event that recruitment targets are not met, sampling will move into the second geographic perimeter. This strategy will maximise the likelihood of recruiting children who have received their vaccines at the selected health centre, minimising recruitment from buffer zonal borders and therefore minimizing risk of contamination (*i.e.* recruitment of children receiving a PCV13 vaccine schedule different than the schedule assigned to the child's local health centre).

ii) Children 5-10 years of age, PCV13-vaccinated with the 3+0 schedule. These children, having received PCV13 at infancy, will be recruited from government schools. Evaluation within this age group will allow us to evaluate for a secondary objective, evaluating the indirect effect of a 2+1 schedule on VT pneumococcal carriage among children aged 5-10 years, the majority of whom received PCV13 infancy EPI vaccination using a 3+0 schedule. Six schools will be selected, three located centrally in each of the 3+0 clusters and the 2+1 clusters. Children will be chosen at random from school registers. This component will allow the evaluation of indirect effects of the 2+1 compared to 3+0 schedule among children with waning vaccine-induced immunity.

iii) Adults 18 - 40 years of age, HIV-infected on ART and PCV13-unvaccinated. Evaluation within group will allow us to achieve the secondary objective of evaluating the indirect effect of a 2+1 schedule on VT pneumococcal carriage among HIV-infected adults (18-40 years) on ART, a population at high risk of pneumococcal disease. Participants will be recruited from the Queen Elizabeth Central Hospital (QECH), Lighthouse ART Clinic in Blantyre.

Safety. To assess the differential effects of 3 *vs*. 2 primary vaccine doses in the first year of life, an additional carriage survey will be implemented at health centres approximately 9 months after 2+1 implementation. A convenience sample of children aged 9-months will be recruited at health centres providing either the 2+1 or 3+0 schedule. This will allow an evaluation of carriage prevalence among children receiving 3 (according to the 3+0 schedule) *vs*. 2 (according to the 2+1 schedule and presenting for their booster dose) primary doses. Additionally, this additional carriage survey contribute to evaluating the coverage of the 2+1 vaccination schedule and the

For peer review only - http://bmjopen.bmj.com/site/about/guidelines.xhtml

proportion of children receiving all three PCV13 doses at health centres providing the 2+1 schedule.

Inclusion and exclusion criteria. Inclusion criteria for all individuals recruited includes permanent resident in Blantyre District. Among children 15-24 months of age (assigned to either 2+1 or 3+0 schedule), inclusion criteria additionally include: aged between 15-24-month, parent/legal guardian providing written informed consent, evidence (recorded in health passport) of having received a full schedule of PCV13. Among children 5-10 years of age, inclusion criteria include: aged between 5-10 years, parent/legal guardian providing written informed consent, providing written informed assent (If the child is aged ≥8), and either verbal or documented evidence of having received PCV13. Among adults, inclusion criteria include: aged 18-40 years, providing written informed consent, and being HIV-infected and receiving ART.

Among children 9 months of age, recruited as part of the safety component, inclusion criteria include: aged 9 months, parent/legal guardian providing written informed consent, and evidence (recorded in health passport) of having received either a full 3+0 PCV13 vaccine schedule (health centres implementing 3+0) or both primary doses of PCV13 at approximately 6 and 14 weeks of age (health centres implementing 2+1).

Exclusion criteria for all screened individuals include receiving TB treatment at time of screening, hospitalization for pneumonia within 14 days prior to study screening and terminal illness. Exclusion criteria for all children include parental/legal guardian not providing consent, not providing assent (for children aged ≥8 years), having received antibiotic treatment within 14 days prior to study screening. Exclusion criteria for adults include not providing written informed consent or prior vaccination with a pneumococcal vaccine.

Intervention. The intervention will consist of two carriage surveys conducted 18 and 33 months after the 10 randomised health centres switch from a 3+0 to a 2+1 PCV13 schedule. In both surveys, a single nasopharyngeal swab will be collected from each participant. Following previously described WHO-recommended procedures, (45,49) these will be collected and taken to the Malawi-Liverpool-Wellcome Trust Clinical

For peer review only - http://bmjopen.bmj.com/site/about/guidelines.xhtml

Research Programme (MLW) laboratory in Blantyre for the isolation and characterization of *S. pneumoniae.* VT pneumococcal carriage will be serotyped by latex agglutination. Samples from participants with confirmed pneumococcal carriage will be sent to the United Kingdom for assessment of multiple serotype carriage (genomic microarray) and for whole genome sequencing (WGS).

Expected Outcomes. The primary endpoint of this study will be the difference in VT pneumococcal carriage prevalence among children aged 15-24 months, comparing those vaccinated with PCV13 in either a 2+1 or 3+0 schedule, three years after implementing the switch to a 2+1 schedule. Additionally, the study will evaluate four secondary outcomes: i) difference in VT carriage prevalence among children 5-10 years old, 18 months and 33 months after 2+1 implementation, ii) VT carriage prevalence among HIV-infected adults aged 18-40 years and receiving ART at the time of sampling, iii) VT carriage prevalence among infants aged 9 months, who will have received 3 primary doses (3+0 health centres) or 2 primary doses (2+1 health centres) prior to the booster dose, 9 months after the implementation of the 2+1 schedule and iv) prevalence of multiple serotype carriage, 18 and 33 months after 2+1 implementation

Statistical Methods

Study Power and Sample Size Calculation. The primary endpoint is powered to detect an effect size of 50% reduction in VT carriage, expecting a 14% and 7% VT carriage prevalence among vaccinated children 15-24 months old in the 3+0 and 2+1 arms, respectively. Sample sizes were calculated based on a power of 80% and a statistical significance of 0.05. The calculations accounted for household similarities using an intra-class correlation (ICC) of 0.005 (based on previous experience in this setting) and adjusted for a design effect (dependent on both ICC and cluster size.) of 1.21. Minimum sample sizes needed to achieve the necessary power under these assumptions are shown in Table 1.

To assess the primary and secondary endpoints i) One child 15-24 months of age (2+1 or 3+0) will be recruited from each of 40 households randomly selected within each of the 20 clusters. This is a total 800 vaccinated children per survey (1,600 total for two surveys). ii) 125 children will be recruited from each of the 6 schools per survey. This

For peer review only - http://bmjopen.bmj.com/site/about/guidelines.xhtml

is a total 750 children per survey (1,500 total). iii) A total of 1,200 HIV-infected adults will be recruited from the QECH ART Clinic over the course of the 3-year study period. iv) A total of 800 children 9 months of age will be recruited from the vaccination centres 9 months after schedule change (800 total).

Data Collection, Management, and Anonymization Procedures. Demographic data and relevant medical history will be collected using password-protected electronic data capturing (EDC). Each participant will be assigned unique participant identification number (PID) at recruitment. This PID will be used in all datasheets and files, and will be linked to the laboratory data, hence, only anonymised data will be used for the analysis. Fully anonymized data will be uploaded daily to a secured on-site server, which is backed up daily to both local and off-site facilities. A logbook containing identifiable information (including name) will be kept separate in a secured location by an authorised member of the study team and will only be accessed by authorised study members. This will allow the study team to recover any missing epidemiological information at a later date (*e.g.* missing vaccination dates) and to facilitate any participants who choose to withdraw consent at any time.

Statistical Analyses

Continuous variables will be summarised by means and standard deviations, or medians and interquartile ranges if the distribution exhibits skew. Categorical variables will be summarised by frequency distributions. Direct effects of the PCV13 schedule change on VT carriage will be ascertained by comparing carriage prevalence in children aged 15-24 months residing in the recruitment clusters of health centres randomised to the 2+1 schedule and those in recruitment clusters of health centres randomised to the 3+0 schedule. Indirect effects will be evidenced by comparing children in the recruitment clusters of health centres randomised to the 3+0 schedule and older children (5-10 years) and HIV+ adults. Additionally, data obtained in this study will be compared to those obtained from previous carriage surveys to ascertain any changes in VT carriage prevalence before and after the PVC13 schedule change. Statistical tests will be selected depending on the distribution patterns of the data.

Ethics and Dissemination

For peer review only - http://bmjopen.bmj.com/site/about/guidelines.xhtml

Ethics Approvals. The study has been approved by the Malawi College of Medicine Research Ethics Committee (COMREC; Ref: P05.19.2680), the University College London Research Ethics Committee (Ref: 8603.002) and the University of Liverpool Research Ethics Committee (Ref: 5439).

Data Monitoring External Advisory Group. The implementation of the study protocol will be reviewed and monitored by an External Advisory Group, providing oversight of the study activities and advise the study team at the time of the interim and final analyses. The group will include experts from the University of Malawi, College of Medicine, the London School of Hygiene & Tropical Medicine, and the Medical Research Council.

Interim Analysis and Changes to Public Health Policy. Upon completion of the first carriage survey, an interim analysis will be implemented using data obtained on the VT carriage prevalence among vaccinated children 15-24 months of age. These results will be used to assess possible adaptation of the second community carriage survey. Three possible scenarios are considered: i) If convincing evidence of major change in carriage prevalence is demonstrated, immediate action will be discussed with the MoH to move to change the schedule and adapt year-3 sampling; ii) If no major change in carriage prevalence is identified, the study will continue as planned; iii) If carriage prevalence has fallen by more than 30% but does not meet the primary threshold of 50%, the second carriage survey will be brought forward by 6 months. Decisions will be reached in consensus between investigators, the Expert Advisory Board, and the MoH.

Informed Consent Process. The study will only recruit children whose parents/legal guardians have the capacity to provide informed consent or, in the case of adults, are capable of giving consent. Children who are minors but ≥8 years of age will be required to provide informed assent, in addition to parents/legal guardians providing informed consent. Participants will receive both verbal and written information about the study and will be given the opportunity to ask questions and express their doubts and concerns before accepting to take part. They will also be given time to reflect before they come to a decision. An informed consent and/or assent form will be signed and dated by the participant and a member of the research team. The participant will keep

For peer review only - http://bmjopen.bmj.com/site/about/guidelines.xhtml

a copy of the document, and a second one will be kept in the study file with the Principal Investigator based in Blantyre, Malawi. Participants will be informed of their right to withdraw consent at any point until study ends without the need to provide a reason, and without penalty.

Dissemination Policy and Plans

Study results will be shared with local stakeholders and published in peer-reviewed journals. Partial results and interim analyses will be shared with the Malawi MoH and other relevant policymakers and decision-making stakeholders. Partial and final findings will be presented at relevant international conferences and meetings. Copies of all published materials and reports will be shared with the research ethics committees (RECs) and collaborators. We will return to the community partners and work with the community advisory board to report and further disseminate our results into those communities where we worked.

Data availability. Data used in analyses for reported findings of this study will be made publicly available on FigShare (https://figshare.com), an online open access repository.

Patient and Public Involvement Statement. Prior to development of the protocol, key stakeholders were informed of the study, including the study sites (*i.e.* selected health centres) and their surrounding communities (*i.e.* catchment areas), the District Health Office (DHO), Ministry of Health and the Ministry of Education. We actively sought and incorporated input from these stakeholders into the study objectives and overall design. Community sensitisation will be further strengthened through a community advisory board.

Acknowledgements. The authors thank the MLW laboratory management team, led by Brigitte Denis and George Selemani.

For peer review only

References

1. Heinsbroek E, Tafatatha T, Phiri A, Swarthout TD, Alaerts M, Crampin AC, et al. Pneumococcal carriage in households in Karonga District, Malawi, before and after introduction of 13-valent pneumococcal conjugate vaccination. Vaccine. 2018 Oct 20;

2. GBD 2016 Lower Respiratory Infections Collaborators. Estimates of the global, regional, and national morbidity, mortality, and aetiologies of lower respiratory infections in 195 countries, 1990-2016: a systematic analysis for the Global Burden of Disease Study 2016. Lancet Infect Dis. 2018;18(11):1191–210.

3. Gray BM, Turner ME, Dillon HC. Epidemiologic studies of Streptococcus pneumoniae in infants. The effects of season and age on pneumococcal acquisition and carriage in the first 24 months of life. Am J Epidemiol. 1982 Oct;116(4):692–703.

4. Ghaffar F, Friedland IR, McCracken GH. Dynamics of nasopharyngeal colonization by Streptococcus pneumoniae. Pediatr Infect Dis J. 1999 Jul;18(7):638–46.

5. Högberg L, Geli P, Ringberg H, Melander E, Lipsitch M, Ekdahl K. Age- and Serogroup-Related Differences in Observed Durations of Nasopharyngeal Carriage of Penicillin-Resistant Pneumococci. J Clin Microbiol. 2007 Mar 1;45(3):948–52.

6. Usuf E, Bottomley C, Adegbola RA, Hall A. Pneumococcal carriage in sub-Saharan Africa--a systematic review. PloS One. 2014;9(1):e85001.

7. Vallès X, Flannery B, Roca A, Mandomando I, Sigaúque B, Sanz S, et al. Serotype distribution and antibiotic susceptibility of invasive and nasopharyngeal isolates of Streptococcus pneumoniae among children in rural Mozambique. Trop Med Int Health TM IH. 2006 Mar;11(3):358–66.

8. Hill PC, Townend J, Antonio M, Akisanya B, Ebruke C, Lahai G, et al. Transmission of Streptococcus pneumoniae in rural Gambian villages: a longitudinal study. Clin Infect Dis Off Publ Infect Dis Soc Am. 2010 Jun 1;50(11):1468–76.

9. Abdullahi O, Karani A, Tigoi CC, Mugo D, Kungu S, Wanjiru E, et al. Rates of acquisition and clearance of pneumococcal serotypes in the nasopharynges of children in Kilifi District, Kenya. J Infect Dis. 2012 Oct 1;206(7):1020–9.

10. Mackenzie GA, Hill PC, Jeffries DJ, Hossain I, Uchendu U, Ameh D, et al. Effect of the introduction of pneumococcal conjugate vaccination on invasive pneumococcal disease in The Gambia: a population-based surveillance study. Lancet Infect Dis. 2016;16(6):703–11.

11. von Gottberg A, de Gouveia L, Tempia S, Quan V, Meiring S, von Mollendorf C, et al. Effects of Vaccination on Invasive Pneumococcal Disease in South Africa. N Engl J Med. 2014 Nov 13;371(20):1889–99.

For peer review only - http://bmjopen.bmj.com/site/about/guidelines.xhtml

12. Hammitt LL, Etyang AO, Morpeth SC, Ojal J, Mutuku A, Mturi N, et al. Effect of ten-valent pneumococcal conjugate vaccine on invasive pneumococcal disease and nasopharyngeal carriage in Kenya: a longitudinal surveillance study. The Lancet. 2019 May 25;393(10186):2146–54.

13. Roca A, Bojang A, Bottomley C, Gladstone RA, Adetifa JU, Egere U, et al. Effect on nasopharyngeal pneumococcal carriage of replacing PCV7 with PCV13 in the Expanded Programme of Immunization in The Gambia. Vaccine. 2015 Dec 16;33(51):7144–51.

14. Adebanjo T, Lessa FC, Mucavele H, Moiane B, Chauque A, Pimenta F, et al. Pneumococcal carriage and serotype distribution among children with and without pneumonia in Mozambique, 2014-2016. PloS One. 2018;13(6):e0199363.

15. Flasche S, Van Hoek AJ, Sheasby E, Waight P, Andrews N, Sheppard C, et al. Effect of pneumococcal conjugate vaccination on serotype-specific carriage and invasive disease in England: a cross-sectional study. PLoS Med. 2011 Apr;8(4):e1001017.

16. Jayasinghe S, Menzies R, Chiu C, Toms C, Blyth CC, Krause V, et al. Long-term Impact of a “3 + 0” Schedule for 7- and 13-Valent Pneumococcal Conjugate Vaccines on Invasive Pneumococcal Disease in Australia, 2002–2014. Clin Infect Dis. 2017 Jan 15;64(2):175–83.

17. Moore MR, Link-Gelles R, Schaffner W, Lynfield R, Lexau C, Bennett NM, et al. Effect of use of 13-valent pneumococcal conjugate vaccine in children on invasive pneumococcal disease in children and adults in the USA: analysis of multisite, population-based surveillance. Lancet Infect Dis. 2015 Mar;15(3):301–9.

18. Prato R, Fortunato F, Cappelli MG, Chironna M, Martinelli D. Effectiveness of the 13-valent pneumococcal conjugate vaccine against adult pneumonia in Italy: a case-control study in a 2-year prospective cohort. BMJ Open. 2018 25;8(3):e019034.

19. Rinta-Kokko H, Palmu AA, Auranen K, Nuorti JP, Toropainen M, Siira L, et al. Long-term impact of 10-valent pneumococcal conjugate vaccination on invasive pneumococcal disease among children in Finland. Vaccine. 2018 05;36(15):1934–40.

20. Ruiz-Contreras J, Picazo J, Casado-Flores J, Baquero-Artigao F, Hernández-Sampelayo T, Otheo E, et al. Impact of 13-valent pneumococcal conjugate vaccine on pneumococcal meningitis in children. Vaccine. 2017 Aug 16;35(35, Part B):4646–51.

21. Waight PA, Andrews NJ, Ladhani SN, Sheppard CL, Slack MPE, Miller E. Effect of the 13-valent pneumococcal conjugate vaccine on invasive pneumococcal disease in England and Wales 4 years after its introduction: an observational cohort study. Lancet Infect Dis. 2015 May;15(5):535–43.

For peer review only - http://bmjopen.bmj.com/site/about/guidelines.xhtml

22. Wiese AD, Griffin MR, Grijalva CG. Impact of pneumococcal conjugate vaccines on hospitalizations for pneumonia in the United States. Expert Rev Vaccines. 2019 Feb 13;0(ja):null.

23. Wiese AD, Huang X, Yu C, Mitchel EF, Kyaw MH, Griffin MR, et al. Changes in otitis media episodes and pressure equalization tube insertions among young children following introduction of the 13-valent pneumococcal conjugate vaccine: A birth-cohort based study. Clin Infect Dis Off Publ Infect Dis Soc Am. 2019 Feb 16;

24. Miller E, Andrews NJ, Waight PA, Slack MP, George RC. Herd immunity and serotype replacement 4 years after seven-valent pneumococcal conjugate vaccination in England and Wales: an observational cohort study. Lancet Infect Dis. 2011 Oct;11(10):760–8.

25. O'Brien KL, Dagan R. The potential indirect effect of conjugate pneumococcal vaccines. Vaccine. 2003 May 16;21(17–18):1815–25.

26. Whitney CG, Farley MM, Hadler J, Harrison LH, Bennett NM, Lynfield R, et al. Decline in invasive pneumococcal disease after the introduction of protein-polysaccharide conjugate vaccine. N Engl J Med. 2003 May 1;348(18):1737–46.

27. Melegaro A, Edmunds WJ. Cost-effectiveness analysis of pneumococcal conjugate vaccination in England and Wales. Vaccine. 2004 Oct 22;22(31–32):4203–14.

28. Simell B, Auranen K, Käyhty H, Goldblatt D, Dagan R, O'Brien KL, et al. The fundamental link between pneumococcal carriage and disease. Expert Rev Vaccines. 2012 Jul;11(7):841–55.

29. Auranen K, Rinta-Kokko H, Goldblatt D, Nohynek H, O'Brien KL, Satzke C, et al. Colonisation endpoints in Streptococcus pneumoniae vaccine trials. Vaccine. 2013 Dec 17;32(1):153–8.

30. Kobayashi M, Conklin LM, Bigogo G, Jagero G, Hampton L, Fleming-Dutra KE, et al. Pneumococcal carriage and antibiotic susceptibility patterns from two cross-sectional colonization surveys among children aged <5 years prior to the introduction of 10-valent pneumococcal conjugate vaccine - Kenya, 2009-2010. BMC Infect Dis. 2017 05;17(1):25.

31. Nunes MC, Shiri T, van Niekerk N, Cutland CL, Groome MJ, Koen A, et al. Acquisition of Streptococcus pneumoniae in pneumococcal conjugate vaccine-naïve South African children and their mothers. Pediatr Infect Dis J. 2013 May;32(5):e192-205.

32. Usuf E, Badji H, Bojang A, Jarju S, Ikumapayi UN, Antonio M, et al. Pneumococcal carriage in rural Gambia prior to the introduction of pneumococcal conjugate vaccine: a population-based survey. Trop Med Int Health TM IH. 2015 Jul;20(7):871–9.

33. Verani JR, Massora S, Acácio S, Santos RT dos, Vubil D, Pimenta F, et al. Nasopharyngeal carriage of Streptococcus pneumoniae among HIV-infected

For peer review only - http://bmjopen.bmj.com/site/about/guidelines.xhtml

and –uninfected children <5 years of age before introduction of pneumococcal conjugate vaccine in Mozambique. PLOS ONE. 2018 Feb 15;13(2):e0191113.

34. Bar-Zeev N, Swarthout TD, Everett DB, Alaerts M, Msefula J, Brown C, et al. Impact and Effectiveness of 13-Valent Pneumococcal Conjugate Vaccine on Population Incidence of Vaccine and Non-Vaccine Serotype Invasive Pneumococcal Disease in Blantyre, Malawi, 2006-2018: Prospective Observational Time-Series and Case-Control Studies [Internet]. Rochester, NY: Social Science Research Network; 2020 Dec [cited 2021 Jan 27]. Report No.: ID 3745169. Available from: https://papers.ssrn.com/abstract=3745169

35. King C, Bar-Zeev N, Phiri T, Beard J, Mvula H, Crampin A, et al. Population impact and effectiveness of sequential 13-valent pneumococcal conjugate and monovalent rotavirus vaccine introduction on infant mortality: prospective birth cohort studies from Malawi. BMJ Glob Health. 2020 Sep;5(9).

36. Cutts FT, Zaman SMA, Enwere G, Jaffar S, Levine OS, Okoko JB, et al. Efficacy of nine-valent pneumococcal conjugate vaccine against pneumonia and invasive pneumococcal disease in The Gambia: randomised, double-blind, placebo-controlled trial. Lancet Lond Engl. 2005 Apr 26;365(9465):1139–46.

37. Kwambana-Adams B, Hanson B, Worwui A, Agbla S, Foster-Nyarko E, Ceesay F, et al. Rapid replacement by non-vaccine pneumococcal serotypes may mitigate the impact of the pneumococcal conjugate vaccine on nasopharyngeal bacterial ecology. Sci Rep. 2017 15;7(1):8127.

38. Usuf E, Bottomley C, Bojang E, Cox I, Bojang A, Gladstone R, et al. Persistence of nasopharyngeal pneumococcal vaccine serotypes and increase of non-vaccine serotypes among vaccinated infants and their mothers five years after PCV13 introduction in The Gambia. Clin Infect Dis Off Publ Infect Dis Soc Am. 2018 Aug 24;

39. WHO Publication. Pneumococcal vaccines WHO position paper - 2012 - recommendations. Vaccine. 2012 Jul 6;30(32):4717–8.

40. Fleming-Dutra KE, Conklin L, Loo JD, Knoll MD, Park DE, Kirk J, et al. Systematic review of the effect of pneumococcal conjugate vaccine dosing schedules on vaccine-type nasopharyngeal carriage. Pediatr Infect Dis J. 2014 Jan;33 Suppl 2:S152-160.

41. Andrejko K, Hosangadi D, Cohen O, Cohen AL, Althouse B, McIntyre P, et al. WHO Technical Expert Consultation Report on Optimization of PCV Impact: Review of Evidence and Programmatic Considerations to Inform Policy. 2017;29.

42. World Health Organization. Pneumococcal Conjugate Vaccine (PCV) Review of Impact Evidence (PRIME) Summary of Findings from Systematic Review [Internet]. 2017 Oct [cited 2019 Mar 12] p. 215. Available from: https://www.who.int/immunization/sage/meetings/2017/october/3_FULL_PRIME_REPORT_2017Sep26.pdf?ua=1

For peer review only

For peer review only - http://bmjopen.bmj.com/site/about/guidelines.xhtml

43. Mvula H, Heinsbroek E, Chihana M, Crampin AC, Kabuluzi S, Chirwa G, et al. Predictors of Uptake and Timeliness of Newly Introduced Pneumococcal and Rotavirus Vaccines, and of Measles Vaccine in Rural Malawi: A Population Cohort Study. PLoS ONE [Internet]. 2016 May 6 [cited 2019 Mar 12];11(5). Available from: https://www.ncbi.nlm.nih.gov/pmc/articles/PMC4859501/

44. Tsega A, Hausi H, Chriwa G, Steinglass R, Smith D, Valle M. Vaccination coverage and timely vaccination with valid doses in Malawi. Vaccine Rep. 2016 Dec 1;6:8–12.

45. Swarthout TD, Fronterre C, Lourenço J, Obolski U, Gori A, Bar-Zeev N, et al. High residual carriage of vaccine-serotype Streptococcus pneumoniae after introduction of pneumococcal conjugate vaccine in Malawi. Nat Commun. 2020 May 6;11(1):2222.

46. Lourenço J, Obolski U, Swarthout TD, Gori A, Bar-Zeev N, Everett D, et al. Determinants of high residual post-PCV13 pneumococcal vaccine-type carriage in Blantyre, Malawi: a modelling study. BMC Med. 2019 Dec 5;17(1):219.

47. National Statistical Office. Government of Malawi. 2018 Malawi Population and Housing Census Main Report [Internet]. Zomba. Malawi: Government of Malawi; 2019 [cited 2019 Nov 8]. Available from: https://malawi.unfpa.org/sites/default/files/resource-pdf/2018%20Malawi%20Population%20and%20Housing%20Census%20Main%20Report%20%281%29.pdf

48. Loudon K, Treweek S, Sullivan F, Donnan P, Thorpe KE, Zwarenstein M. The PRECIS-2 tool: designing trials that are fit for purpose. BMJ. 2015 May 8;350:h2147.

49. Satzke C, Turner P, Virolainen-Julkunen A, Adrian PV, Antonio M, Hare KM, et al. Standard method for detecting upper respiratory carriage of Streptococcus pneumoniae: updated recommendations from the World Health Organization Pneumococcal Carriage Working Group. Vaccine. 2013 Dec 17;32(1):165–79.

Contributors TDS, GK, CM, NF, and RHS conceived the study. TDS, GK, CM, NF, and RHS designed the study with contributions from AI, JK, MB, MYRH, SG,. TDS and CB and JM oversaw development of laboratory methods. TDS, AI, PC and GS designed study documents. TDS and AI jointly wrote the first draft. All authors have commented on the manuscript. All authors have read and approved the final manuscript.

Competing interest. The authors declare that they have no competing interests.

For peer review only - http://bmjopen.bmj.com/site/about/guidelines.xhtml

Funding This work was funded by Bill & Melinda Gates Foundation, USA (Ref: OPP1185516) to RSH; National Institute for Health Research (NIHR) Global Health Research Unit on Mucosal Pathogens using UK aid from the UK Government (Project number 16/136/46) to RSH. The MLW Clinical Research Programme is supported by a Strategic Award from the Wellcome Trust, UK (206545/Z/17/Z) to SG

For peer review only

For peer review only - http://bmjopen.bmj.com/site/about/guidelines.xhtml

Figure Legends

Figure 1. Map of Blantyre District with borders of health centre catchment areas Red crosses (+) indicate location of health centres offering EPI vaccination. Areas shaded in green are non-inhabited (incl. mountains, industrial zones, and other regions administratively declared not for habitation).

Figure 2. The PRagmatic-Explanatory Continuum Indicator Summary 2 (PRECIS-2) wheel with study-specific scores.

Figure 3. Carriage surveillance sampling frame Black dashed vertical line separates primary (left of dashed line) from secondary study objectives. QECH=Queen Elizabeth Central Hospital, PCV=pneumococcal conjugate vaccine, ART=anti-retroviral, m=months, yrs=years

For peer review only

Table 1: Sample size estimations

N per cluster	ICC	p1	p2	power	alpha	DE	N per arm	Clusters, per arm	Clusters, total	N total
40	0.005	0.14	0.07	0.8	0.05	1.20	358	9	18	720
40	0.005	0.14	0.07	0.8	0.05	1.21	400	10	20	800
45	0.005	0.14	0.07	0.8	0.05	1.22	366	9	18	810
50	0.005	0.14	0.07	0.8	0.05	1.25	373	8	16	800
55	0.005	0.14	0.07	0.8	0.05	1.27	381	7	14	770
60	0.005	0.14	0.07	0.8	0.05	1.30	388	7	14	840
40	0.01	0.14	0.07	0.8	0.05	1.39	417	11	22	880
45	0.01	0.14	0.07	0.8	0.05	1.44	432	10	20	900
50	0.01	0.14	0.07	0.8	0.05	1.49	447	9	18	900
55	0.01	0.14	0.07	0.8	0.05	1.54	462	9	18	990
60	0.01	0.14	0.07	0.8	0.05	1.59	477	8	16	960
40	0.015	0.14	0.07	0.8	0.05	1.59	475	12	24	960

DE=design effect, N=number of children recruited, ICC=intra-class correlation

For peer review only

For peer review only - http://bmjopen.bmj.com/site/about/guidelines.xhtml

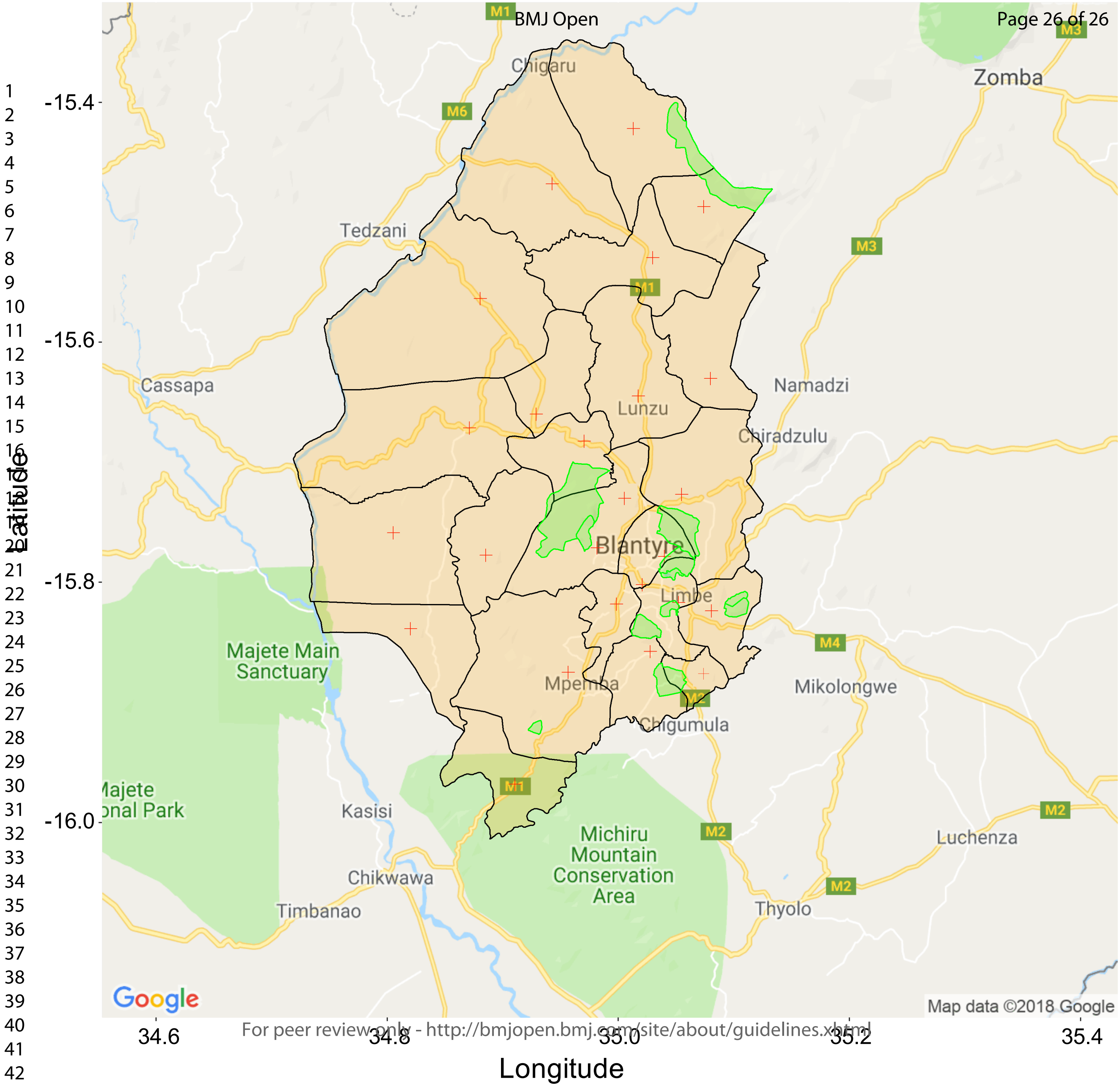
Chigaru
Zomba
Tedzani
Cassapa
Namadzi
Lunzu
Chiradzulu
Blantyre
Limbe
Majete Main
Sanctuary
Mpemba
Mikolongwe
Chigumula
Kasisi
Michiru
Mountain
Conservation
Area
Luchenza
Chikwawa
Timbanao
Thyolo
M1
M2
M3
M4
M6
Google
Map data ©2018 Google
-15.4
-15.6
-15.8
-16.0
34.6
34.8
35.0
35.2
35.4
Latitude
Longitude

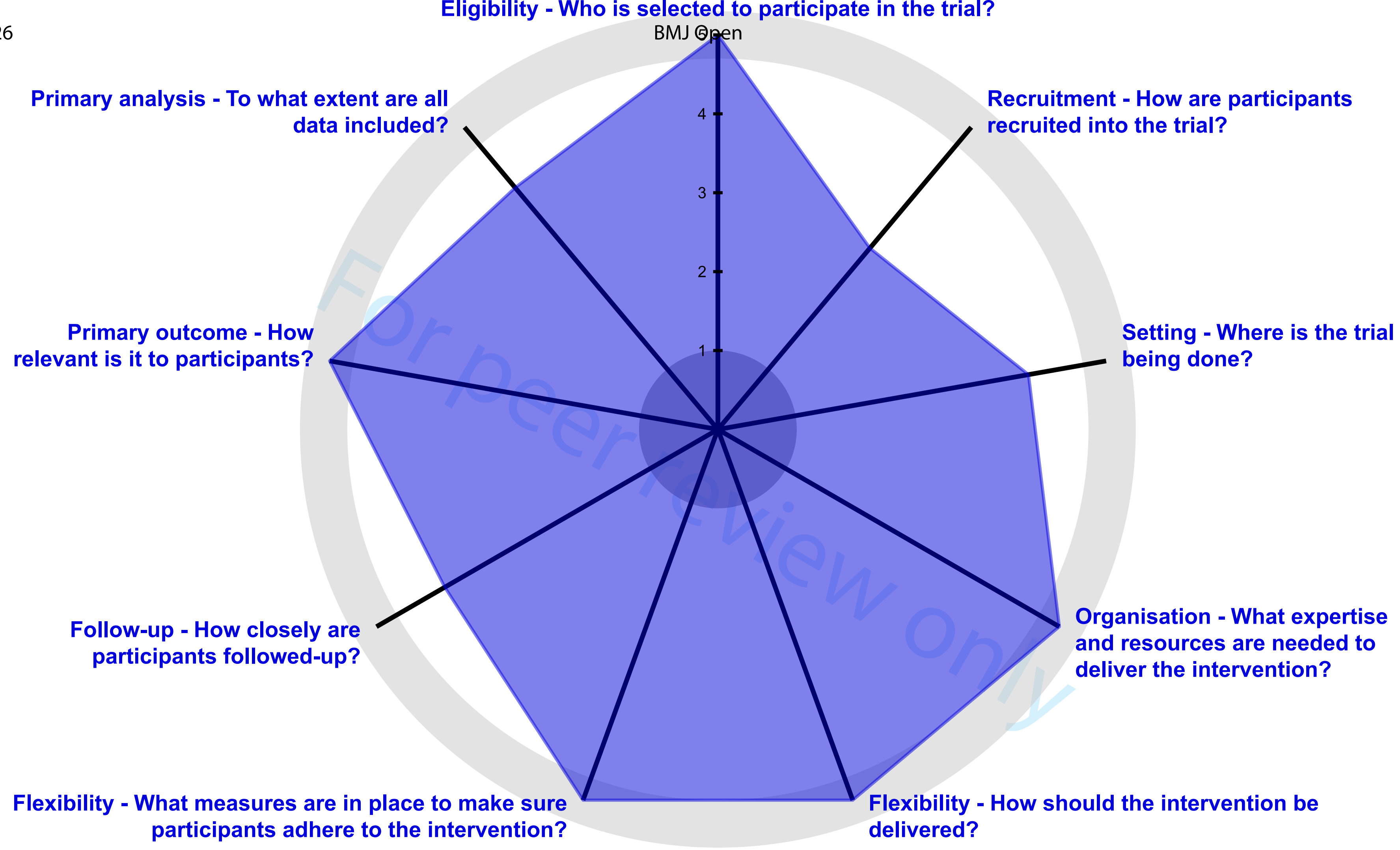

Eligibility	Recruitment	Setting	Organisation	Flexibility (delivery)	Flexibility (Adhere)	Follow-up	Primary outcome	Primary analysis
5	3	4	5	5	5	4	5	4

Surveillance Sampling Frame

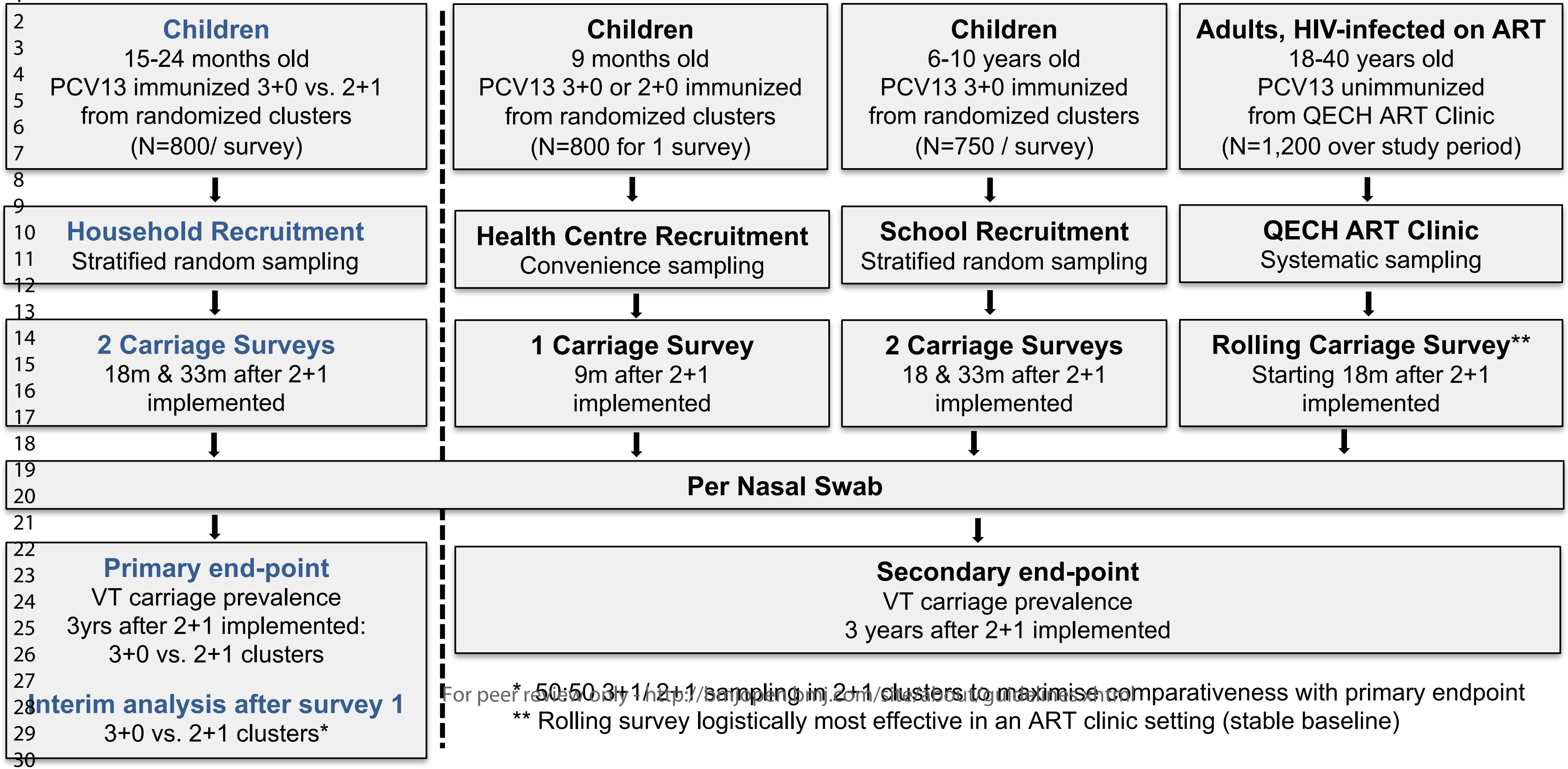

For peer review only - http://bmjopen.bmj.com/site/about/guidelines.xhtml

BMJ Open

A pragmatic health centre-based evaluation comparing the effectiveness of a PCV13 schedule change from 3+0 to 2+1 in a high pneumococcal carriage and disease burden setting in Malawi: a study protocol

Journal:	*BMJ Open*
Manuscript ID	bmjopen-2021-050312.R1
Article Type:	Protocol
Date Submitted by the Author:	20-Apr-2021

Complete List of Authors:	Swarthout, Todd; Malawi-Liverpool-Wellcome Trust Clinical Research Programme; University College London, Division of Infection and Immunity<br>Ibarz-Pavon, Ana; Malawi-Liverpool-Wellcome Trust Clinical Research Programme; University of Liverpool<br>Kawalazira, Gift; Ministry of Health<br>Sinjani, George; Malawi-Liverpool-Wellcome Trust Clinical Research Programme<br>Chirombo, James; Malawi-Liverpool-Wellcome Trust Clinical Research Programme<br>Gori, Andrea; University College London<br>Chalusa, Peter; Malawi-Liverpool-Wellcome Trust Clinical Research Programme<br>Bonomali, Farouck; Malawi-Liverpool-Wellcome Trust Clinical Research Programme<br>Nyirenda, Roseline; Malawi-Liverpool-Wellcome Trust Clinical Research Programme<br>Bulla, Edwin; Malawi-Liverpool-Wellcome Trust Clinical Research Programme<br>Brown, Comfort; Malawi-Liverpool-Wellcome Trust Clinical Research Programme<br>Msefula, Jacquline; Malawi-Liverpool-Wellcome Trust Clinical Research Programme<br>Banda, Marjory; Ministry of Education<br>Kachala, Jean; Ministry of Health<br>Mwansambo, Charles; Ministry of Health<br>Henrion, Marc; Malawi Liverpool Wellcome Trust Clinical Research Programme; Liverpool School of Tropical Medicine, Department of Clinical Sciences<br>Gordon, Stephen; University of Malawi College of Medicine, Malawi-Liverpool-Wellcome Trust Clinical Research Programme; Liverpool School of Tropical Medicine Department of Clinical Sciences, Clinical Sciences<br>French, Neil; University of Liverpool Faculty of Health and Life Sciences, Centre for Global Vaccine Research, Institute of Infection and Global Health; University of Malawi College of Medicine, Malawi-Liverpool-Wellcome Trust Clinical Research Programme<br>Heyderman, Robert; University College London, Division of Infection and Immunity; University of Malawi College of Medicine, Malawi Liverpool

	Wellcome Trust Clinical Research Programme
**Primary Subject Heading**:	Infectious diseases
Secondary Subject Heading:	Epidemiology, Public health
Keywords:	EPIDEMIOLOGY, Epidemiology < INFECTIOUS DISEASES, Public health < INFECTIOUS DISEASES, Paediatric infectious disease & immunisation < PAEDIATRICS, PUBLIC HEALTH

SCHOLARONE™
Manuscripts

I, the Submitting Author has the right to grant and does grant on behalf of all authors of the Work (as defined in the below author licence), an exclusive licence and/or a non-exclusive licence for contributions from authors who are: i) UK Crown employees; ii) where BMJ has agreed a CC-BY licence shall apply, and/or iii) in accordance with the terms applicable for US Federal Government officers or employees acting as part of their official duties; on a worldwide, perpetual, irrevocable, royalty-free basis to BMJ Publishing Group Ltd ("BMJ") its licensees and where the relevant Journal is co-owned by BMJ to the co-owners of the Journal, to publish the Work in this journal and any other BMJ products and to exploit all rights, as set out in our licence.

The Submitting Author accepts and understands that any supply made under these terms is made by BMJ to the Submitting Author unless you are acting as an employee on behalf of your employer or a postgraduate student of an affiliated institution which is paying any applicable article publishing charge ("APC") for Open Access articles. Where the Submitting Author wishes to make the Work available on an Open Access basis (and intends to pay the relevant APC), the terms of reuse of such Open Access shall be governed by a Creative Commons licence – details of these licences and which Creative Commons licence will apply to this Work are set out in our licence referred to above.

Other than as permitted in any relevant BMJ Author's Self Archiving Policies, I confirm this Work has not been accepted for publication elsewhere, is not being considered for publication elsewhere and does not duplicate material already published. I confirm all authors consent to publication of this Work and authorise the granting of this licence.

For peer review only

A pragmatic health centre-based evaluation comparing the effectiveness of a PCV13 schedule change from 3+0 to 2+1 in a high pneumococcal carriage and disease burden setting in Malawi: a study protocol

Todd D. Swarthout[1,2,¥,#], Ana Ibarz-Pavon[1,3¥], Gift Kawalazira[4], George Sinjani[1], James Chirombo[1], Andrea Gori[2], Peter Chalusa[1], Farouck Bonomali[1], Roseline Nyirenda[1], Edwin Bulla[1], Comfort Brown[1], Jacquline Msefula[1], Marjory Banda[5], Jean Kachala[4], Charles Mwansambo[4], Marc Y.R. Henrion[1,3], Stephen B Gordon[1], Neil French[1,4*], Robert S. Heyderman[1,2*]

Author Affiliations

[1]Malawi-Liverpool-Wellcome Trust Clinical Research Programme, Blantyre, Malawi

[2]NIHR Global Health Research Unit on Mucosal Pathogens, Division of Infection and Immunity, University College London, London, United Kingdom

[3]Department of Clinical Sciences, Liverpool School of Tropical Medicine, Liverpool, United Kingdom

[4]Centre for Global Vaccine Research, Institute of Infection, Veterinary, and Ecological Science, University of Liverpool, Liverpool, United Kingdom

[4]Ministry of Health, Lilongwe, Malawi

[5]Ministry of Education, Blantyre, Malawi

¥Todd D. Swarthout and Ana Ibarz-Pavon have contributed equally to this manuscript

* Neil French and Robert Heyderman have contributed equally to this manuscript

#Corresponding author: Todd Swarthout, Malawi-Liverpool-Wellcome Trust Clinical Research Programme, P.O. Box 30096, Blantyre, Malawi; Email: t.swarthout@ucl.ac.uk; Phone: +265 (0)1 874 628

For peer review only

Abstract

Introduction. *Streptococcus pneumoniae* (the pneumococcus) is commonly carried as a commensal bacterium in the nasopharynx but can cause life-threatening disease. Transmission occurs by human respiratory droplets and interruption of this process provides herd immunity. A 2017 WHO Consultation on Optimization of PCV Impact highlighted a substantial research gap in investigating why the impact of PCV vaccines

in low-income countries has been lower than expected. Malawi introduced the 13-valent PCV (PCV13) into the national Expanded Program of Immunizations (EPI) in 2011, using a 3+0 (3 primary + 0 booster doses) schedule. With evidence of greater impact of a 2+1 (2 primary + 1 booster dose) schedule in other settings, including South Africa, Malawi's National Immunizations Technical Advisory Group is seeking evidence of adequate superiority of a 2+1 schedule to inform vaccine policy.

Methods. A pragmatic health centre-based evaluation comparing impact of a PCV13 schedule change from 3+0 to 2+1 in Blantyre District, Malawi. Twenty government health centres will be randomly selected, with ten implementing a 2+1 and ten to continue with the 3+0 schedule. Health centres implementing 3+0 will serve as the direct comparator in evaluating 2+1 providing superior direct and indirect protection against pneumococcal carriage. Pneumococcal carriage surveys will evaluate carriage prevalence among children 15-24 months, randomized at household level, and schoolgoers 5-10 years of age, randomly selected from school registers. Carriage surveys will be conducted 18 and 33 months following 2+1 implementation.

Analysis. The primary endpoint is powered to detect an effect size of 50% reduction in VT carriage among vaccinated children 15-24 months old, expecting a 14% and 7% VT carriage prevalence in the 3+0 and 2+1 arms, respectively.

Ethics and dissemination. The study has been approved by the Malawi College of Medicine Research Ethics Committee (COMREC; Ref: P05.19.2680), the University College London Research Ethics Committee (Ref: 8603.002) and the University of Liverpool Research Ethics Committee (Ref: 5439). The results from this study will be actively disseminated through manuscript publications and conference presentations.

Trial Registration number NCT04078997.

Article Summary

Strengths and limitations of this study

- The study is supported by more than 20 years of routine pneumococcal surveillance.
- The study design is both pragmatic and robust, offering a methodology that is relevant to diverse settings in the region.

For peer review only

For peer review only - http://bmjopen.bmj.com/site/about/guidelines.xhtml

- By engaging community, institutional and government partners, we have optimised its relevance and increased the likelihood of community uptake.
- Limitations to this study include a risk of contamination between clusters (*i.e.* children receiving a 2+1 vaccine schedule who reside in or relocate to a cluster implementing the 3+0 vaccine schedule, or *vice-versa*).

Key Words: *Streptococcus pneumoniae*; immunity, herd; 13-valent pneumococcal vaccine; Africa; Malawi; pneumococcal vaccines; immunization; schedule

For peer review only

For peer review only - http://bmjopen.bmj.com/site/about/guidelines.xhtml

Introduction

Streptococcus pneumoniae, the pneumococcus, is commonly carried as a commensal bacterium in the nasopharynx but can cause life-threatening disease. Infections due to the pneumococcus are estimated to be responsible for approximately 300,000 deaths worldwide, with one-third of these occurring among children under five years of age and with the greatest burden in low- and middle-income country settings (1,2).

S. pneumoniae has almost 100 serotypes, with nasopharyngeal (NP) carriage as a pre-requisite for the development of disease but also a key process for developing natural immunity (3). Transmission occurs largely *via* human respiratory air droplets. With serotype-specific differences, carriage duration decreases with age, lasting from 2 weeks in adults up to 4 months among children (4,5). Pneumococcal carriage prevalence is age-dependent, peaking among children <5 years. Pneumococcal carriage prevalence reported in sub-Sahara Africa are among the highest described; up to 28% among adults and in excess of 80% in children <5 years old, resulting in high transmission rates (6–14).

In HICs, routine administration of pneumococcal conjugate vaccines (PCV) through the infant immunization schedule has contributed to a rapid decline of vaccine serotype invasive pneumococcal disease (VT-IPD) in both vaccinated and unvaccinated populations (15–23). PCVs protect the vaccinated individual (direct protection) against pneumococcal disease and carriage. The resulting reduction in carriage also interrupts transmission from the vaccinated individual to the unvaccinated population (indirect protection). The resulting herd immunity effect has been a major contributor to the success of vaccination programs (24–26). The added cost-effectiveness and vaccine impact gained through indirect protection have been key drivers of vaccination policy in these settings (27,28). PCV impact on pneumococcal carriage continues to be considered a viable endpoint in vaccine licensure evaluations (29).

Studies undertaken prior to PCV introduction in The Gambia, Kenya, Mozambique, Malawi and South Africa reported VT carriage prevalence ranging from 28% to nearly 50% among children <5 years old (30–33). While PCV introduction in African countries

For peer review only - http://bmjopen.bmj.com/site/about/guidelines.xhtml

has resulted in substantial direct effects in reducing risk of VT-IPD (34), pneumonia and all-cause mortality (35) among vaccinated children (10,11,36), the impact of PCV on VT carriage has been markedly less than that observed in HICs. Although Kenya (12), the Gambia (13), Mozambique (14), and South Africa (31) have reported VT carriage reductions, prevalence remains higher than expected, and serotype replacement (i.e. rise in non-VT (NVT) carriage prevalence associated with decrease in VT carriage) is increasing (6,37). As the impact of PCV on carriage is considered an indicator of vaccine impact (29), it remains uncertain whether PCV introduction in sub-Sahara Africa will achieve the sustained direct or indirect protection necessary to reduce pneumococcal carriage to levels sufficient to interrupt transmission and disease (38).

Currently, the World Health Organization (WHO) recommends the implementation of the PCV vaccine using either a or a 3+0 schedule (three primary doses, most commonly at 6, 10, and 14 weeks of age) or a 2+1 schedule (two primary infancy doses at 6 and 14 weeks of age and one booster at 9 months of age or after the 1st year of life). The WHO further recommends that the decision on which schedule to use be based on the epidemiology of disease in the local setting (39). While both schedules have been shown to be effective in reducing VT disease and VT carriage, there have been no direct comparisons of vaccine impact on carriage in a high burden setting (40).

A 2017 WHO Technical Expert Consultation on Optimization of PCV Impact highlighted a substantial research gap in investigating why the impact of PCV vaccines in low-income countries has been less than expected, underlining the need to define an optimal PCV vaccination schedule that will maximize their benefit in such settings (41). The PCV Review of Impact Evidence (PRIME), commissioned to supplement the WHO Expert Consultation, is a systematic review of available evidence on PCV effectiveness and impact on nasopharyngeal carriage, disease and mortality, as well as on PCV immune response. The review included evidence from research studies published between January 2010-December 2016 recorded in 14 databases. Those attending the 2017 WHO Technical Expert Consultation gave head-to-head studies of 2+1 vs 3+0 vaccine schedules the greatest research priority (42). Countries with a high pneumococcal disease and carriage burden, such as Malawi, could implement a

For peer review only - http://bmjopen.bmj.com/site/about/guidelines.xhtml

2+1 schedule quickly, with limited logistical or financial demands, providing a booster dose at the time of the first measles vaccine at 9 months of age.

Malawi introduced the 13-valent PCV (PCV13) into the national Expanded Program of Immunizations (EPI) in November 2011, using a 3+0 schedule (6, 10 and 14 weeks of age), with a three-dose catch-up vaccination campaign among all infants <1 year of age. This introduction has been highly successful, with field studies showing an EPI vaccine coverage exceeding 90% (43,44). Similar to other settings, vaccine introduction resulted in a ~70% reduction in IPD among PCV-vaccinated children (34) and an estimated 35% fall in all-cause mortality (35). However, high levels of residual VT carriage persist in Malawi among vaccinated children up to eight years after the introduction of the vaccine (45). As presented by Lourenço *et. al*. (46), a high force of infection in settings such as Malawi contribute significantly to a 3+0 schedule achieving only a short duration of VT carriage control in infants. Though a 2+1 schedule, as implemented in South Africa, may improve colonisation control, this remains unproven in other African settings such as Malawi.

In this context, the Malawi Ministry of Health (MoH) and the National Immunizations Technical Advisory Group (NITAG) are now seeking evidence of adequate superiority of a 2+1 vaccine schedule to inform a change to Malawi's current EPI schedule. To this aim, a pragmatic health centre-based evaluation comparing the current 3+0 schedule to a 2+1 schedule will be implemented in Blantyre District, southern Malawi. Two pneumococcal carriage surveys, conducted 18 and 33 months following the implementation of the 2+1 schedule, will have the objective of comparing the effect of the two schedules against carriage reduction among otherwise-healthy children and evaluate their potential to enhance herd immunity.

For peer review only - http://bmjopen.bmj.com/site/about/guidelines.xhtml

Methods and analysis

Study design: The study is a pragmatic health centre-based randomized evaluation of the direct effect of a 2+1 PCV13 vaccine schedule on pneumococcal carriage in vaccinated infants and the indirect effect on non-vaccine eligible children and high-risk adults.

Study setting. The study will be conducted in Blantyre District, southern Malawi. The District is 240 Km^2. Healthcare is delivered through a network of private and government hospitals and 28 government primary health centres, where EPI vaccinations are administered. Queen Elizabeth Central Hospital (QECH) is the government referral hospital providing free medical care to the 1·3 million urban, peri-urban and rural residents of Blantyre District. Children <5 years account for 16% of the total population (47). Health centres cover a fixed geographic area (here referred to as 'clusters'), and study sampling will be undertaken within these clusters (Figure 1).

Study questions. We will investigate whether a 2+1 PCV13 vaccination schedule (2 primary doses at 6 and 14 weeks of age + 1 booster dose at 9 months of age), compared to the current 3+0 schedule (3 primary doses at 6, 10 and 14 weeks of age with no booster), is superior in reducing VT carriage prevalence among vaccinated children and, consequently, creating a superior herd effect among older children and HIV-infected adults. To assess the effect of a district-wide change from 3+0 to 2+1, this study will address two specific research questions: Firstly, will a 2+1 schedule provide enduring vaccine-induced protection against pneumococcal VT carriage into the second year of life? Secondly, will the 2+1 vaccine schedule generate stronger herd protection and result in decreased VT carriage in older children and unvaccinated high-risk adults in the general population?

Primary objective. The primary objective of the study is to evaluate the direct effect of a 2+1 PCV13 vaccination schedule on VT pneumococcal carriage among children aged 15-24 months, three years after introducing the 2+1 schedule. This objective will answer the question of whether the 2+1 schedule induces a more enduring direct

For peer review only - http://bmjopen.bmj.com/site/about/guidelines.xhtml

protection against pneumococcal VT carriage into the second year of life, compared to that observed with a 3+0 schedule.

Secondary objectives. The secondary objectives of the study are to evaluate, three years after introducing the 2+1 schedule, i) the indirect effect of a 2+1 schedule on VT pneumococcal carriage among children aged 5-10 years (PCV age-ineligible at time of implementing the 2+1 schedule; ii) the indirect effect of a 2+1 vaccination schedule on VT pneumococcal carriage among HIV-infected adults 18-40 years old and on antiretroviral therapy (ART).

To address these questions, the Malawi MoH and Blantyre District Health Office (DHO) will randomly select 10 health centres (among a total 28 in Blantyre District) in which the routine PCV13 schedule will be switched to a 2+1 schedule. An additional 10 health centres will be randomly selected to continue with the current 3+0 schedule but will serve as the direct comparator in evaluating the effectiveness of the 2+1 schedule.

A pragmatic study design. This switch to a 2+1 schedule is an initiative led by the MoH and will be implemented within the scope of the routine EPI programme, subject to EPI standard procedures for delivery, monitoring, and performance assessment. Implementation of all vaccination activities will be implemented, as per routine practices, by MoH EPI vaccinators through the routine EPI programme. The MoH will monitor completeness of dosing following standard reporting practices within the scope of the EPI. Routine study activities throughout the duration of the three-year study period will include research nurses providing support and guidance to the EPI vaccination teams through weekly site visits. In addition, research enumerators will monitor patient-retained health passports of a representative sample population of vaccinees to confirm they are receiving the proper vaccine schedule (2+1 or 3+0) assigned to the catchment population of their respective health centre.

The pragmatic design of this study has been assessed through the PRECIS-2 tool (The PRagmatic-Explanatory Continuum Indicator Summary 2) (48). This tool was developed to help trialists make design decisions consistent with the intended purpose of their trial. To facilitate domain discussion and consensus, PRECIS-2

For peer review only - http://bmjopen.bmj.com/site/about/guidelines.xhtml

assesses the pragmatic design through nine domains - eligibility criteria, recruitment, setting, organisation, flexibility (delivery), flexibility (adherence), follow-up, primary outcome, and primary analysis - scored from 1 (very explanatory) to 5 (very pragmatic). The report from this tool is in the format of a wheel (Figure 2). Results (mean score: 4.4 and range: 3-5) supports that this is largely a pragmatic randomised study undertaken in the "real world" and with usual care and is intended to help support a decision on whether to deliver an intervention.

3+1 schedule during early period of initiation. To ensure the 2+1 schedule is implemented efficiently, and to limit the risk of confusion during the transition to the 2+1 schedule (including not deferring PCV13 vaccination to a later visit), a 3+1 schedule will be implemented during the first several months at health centres implementing the 2+1 schedule. At these health centres, children who have received either their 10-week or 14-week PCV13 dose before 2+1 implementation will receive a dose at their 14-week (3rd dose PCV13) and their 40-week (4th dose PCV13). Approximately 6 months after 2+1 implementation, all first-contact vaccine visits (post 2+1 implementation) will be for the scheduled visit at 6-weeks of age and the 3+1 schedule will no longer be required.

Carriage surveys. The study will include two cross-sectional carriage surveys, implemented 18 and 33 months after the switch to 2+1. Carriage surveys will be conducted using a well-established methodology implemented extensively in the setting (45,46), allowing comparability of study results to previous carriage surveys conducted in the area. The sampling frame is illustrated in Figure 3.

Sampling will include:

i) children 15-24 months of age, PCV13-vaccinated (confirmed by reviewing patient-retained health passports) with either the 2+1 or the 3+0 schedule. Evaluation within this age group will allow us to evaluate for our primary objective, evaluating the direct effect of vaccination on carriage. Within each of the catchment areas of the 20 participating health centres, we will conduct a random walk method to systematically identify households with eligible children. A 'egg and yolk' strategy will be applied, defining two geographic sampling perimeters around each health centre. Sampling will prioritize those living within the geographic perimeter closest to the health centre. In

For peer review only

For peer review only - http://bmjopen.bmj.com/site/about/guidelines.xhtml

the event that recruitment targets are not met, sampling will move into the second geographic perimeter. This strategy will maximise the likelihood of recruiting children who have received their vaccines at the selected health centre, minimising recruitment from buffer zonal borders and therefore minimizing risk of contamination (*i.e.* recruitment of children receiving a PCV13 vaccine schedule different than the schedule assigned to the child's local health centre).

ii) Children 5-10 years of age, PCV13-vaccinated with the 3+0 schedule. These children, having received PCV13 at infancy, will be recruited from government schools. Evaluation within this age group will allow us to evaluate for a secondary objective, evaluating the indirect effect of a 2+1 schedule on VT pneumococcal carriage among children aged 5-10 years, the majority of whom received PCV13 infancy EPI vaccination using a 3+0 schedule. Six schools will be selected, three located centrally in each of the 3+0 clusters and the 2+1 clusters. Children will be chosen at random from school registers. This component will allow the evaluation of indirect effects of the 2+1 compared to 3+0 schedule among children with waning vaccine-induced immunity.

iii) Adults 18 - 40 years of age, HIV-infected on ART and PCV13-unvaccinated. Evaluation within group will allow us to achieve the secondary objective of evaluating the indirect effect of a 2+1 schedule on VT pneumococcal carriage among HIV-infected adults (18-40 years) on ART, a population at high risk of pneumococcal disease. Participants will be recruited from the Queen Elizabeth Central Hospital (QECH), Lighthouse ART Clinic in Blantyre.

Safety. To assess the differential effects of 3 *vs.* 2 primary vaccine doses in the first year of life, an additional carriage survey will be implemented at health centres approximately 9 months after 2+1 implementation. A convenience sample of children aged 9-months will be recruited at health centres providing either the 2+1 or 3+0 schedule. This will allow an evaluation of carriage prevalence among children receiving 3 (according to the 3+0 schedule) *vs.* 2 (according to the 2+1 schedule and presenting for their booster dose) primary doses. Additionally, this additional carriage survey contribute to evaluating the coverage of the 2+1 vaccination schedule and the

For peer review only - http://bmjopen.bmj.com/site/about/guidelines.xhtml

proportion of children receiving all three PCV13 doses at health centres providing the 2+1 schedule.

Inclusion and exclusion criteria. Inclusion criteria for all individuals recruited includes permanent resident in Blantyre District. Among children 15-24 months of age (assigned to either 2+1 or 3+0 schedule), inclusion criteria additionally include: aged between 15-24-month, parent/legal guardian providing written informed consent, evidence (recorded in health passport) of having received a full schedule of PCV13. Among children 5-10 years of age, inclusion criteria include: aged between 5-10 years, parent/legal guardian providing written informed consent, providing written informed assent (If the child is aged ≥8), and either verbal or documented evidence of having received PCV13. Among adults, inclusion criteria include: aged 18-40 years, providing written informed consent, and being HIV-infected and receiving ART.

Among children 9 months of age, recruited as part of the safety component, inclusion criteria include: aged 9 months, parent/legal guardian providing written informed consent, and evidence (recorded in health passport) of having received either a full 3+0 PCV13 vaccine schedule (health centres implementing 3+0) or both primary doses of PCV13 at approximately 6 and 14 weeks of age (health centres implementing 2+1).

Exclusion criteria for all screened individuals include receiving TB treatment at time of screening, hospitalization for pneumonia within 14 days prior to study screening and terminal illness. Exclusion criteria for all children include parental/legal guardian not providing consent, not providing assent (for children aged ≥8 years), having received antibiotic treatment within 14 days prior to study screening. Exclusion criteria for adults include not providing written informed consent or prior vaccination with a pneumococcal vaccine.

Intervention. The intervention will consist of two carriage surveys conducted 18 and 33 months after the 10 randomised health centres switch from a 3+0 to a 2+1 PCV13 schedule. In both surveys, a single nasopharyngeal swab will be collected from each participant. Following previously described WHO-recommended procedures, (45,49) these will be collected and taken to the Malawi-Liverpool-Wellcome Trust Clinical

For peer review only - http://bmjopen.bmj.com/site/about/guidelines.xhtml

Research Programme (MLW) laboratory in Blantyre for the isolation and characterization of *S. pneumoniae.* VT pneumococcal carriage will be serotyped by latex agglutination. Samples from participants with confirmed pneumococcal carriage will be sent to the United Kingdom for assessment of multiple serotype carriage (genomic microarray) and for whole genome sequencing (WGS).

Expected Outcomes. The primary endpoint of this study will be the difference in VT pneumococcal carriage prevalence among children aged 15-24 months, comparing those vaccinated with PCV13 in either a 2+1 or 3+0 schedule, three years after implementing the switch to a 2+1 schedule. Additionally, the study will evaluate four secondary outcomes: i) difference in VT carriage prevalence among children 5-10 years old, 18 months and 33 months after 2+1 implementation, ii) VT carriage prevalence among HIV-infected adults aged 18-40 years and receiving ART at the time of sampling, iii) VT carriage prevalence among infants aged 9 months, who will have received 3 primary doses (3+0 health centres) or 2 primary doses (2+1 health centres) prior to the booster dose, 9 months after the implementation of the 2+1 schedule and iv) prevalence of multiple serotype carriage, 18 and 33 months after 2+1 implementation

Patient and Public Involvement Statement. Prior to development of the protocol, key stakeholders were informed of the study, including the study sites (*i.e.* selected health centres) and their surrounding communities (*i.e.* catchment areas), the District Health Office (DHO), Ministry of Health and the Ministry of Education. We actively sought and incorporated input from these stakeholders into the study objectives and overall design. Community sensitisation will be further strengthened through a community advisory board.

Statistical Methods

Study Power and Sample Size Calculation. The primary endpoint is powered to detect an effect size of 50% reduction in VT carriage, expecting a 14% and 7% VT carriage prevalence among vaccinated children 15-24 months old in the 3+0 and 2+1 arms, respectively. Sample sizes were calculated based on a power of 80% and a statistical significance of 0.05. The calculations accounted for household similarities using an intra-class correlation (ICC) of 0.005 (based on previous experience in this

For peer review only - http://bmjopen.bmj.com/site/about/guidelines.xhtml

setting) and adjusted for a design effect (dependent on both ICC and cluster size.) of 1.21. Minimum sample sizes needed to achieve the necessary power under these assumptions are shown in Table 1.

To assess the primary and secondary endpoints i) One child 15-24 months of age (2+1 or 3+0) will be recruited from each of 40 households randomly selected within each of the 20 clusters. This is a total 800 vaccinated children per survey (1,600 total for two surveys). ii) 125 children will be recruited from each of the 6 schools per survey. This is a total 750 children per survey (1,500 total). iii) A total of 1,200 HIV-infected adults will be recruited from the QECH ART Clinic over the course of the 3-year study period. iv) A total of 800 children 9 months of age will be recruited from the vaccination centres 9 months after schedule change (800 total).

Data Collection, Management, and Anonymization Procedures. Demographic data and relevant medical history will be collected using password-protected electronic data capturing (EDC). Each participant will be assigned unique participant identification number (PID) at recruitment. This PID will be used in all datasheets and files, and will be linked to the laboratory data, hence, only anonymised data will be used for the analysis. Fully anonymized data will be uploaded daily to a secured on-site server, which is backed up daily to both local and off-site facilities. A logbook containing identifiable information (including name) will be kept separate in a secured location by an authorised member of the study team and will only be accessed by authorised study members. This will allow the study team to recover any missing epidemiological information at a later date (*e.g.* missing vaccination dates) and to facilitate any participants who choose to withdraw consent at any time.

Statistical Analyses

Continuous variables will be summarised by means and standard deviations, or medians and interquartile ranges if the distribution exhibits skew. Categorical variables will be summarised by frequency distributions. Direct effects of the PCV13 schedule change on VT carriage will be ascertained by comparing carriage prevalence in children aged 15-24 months residing in the recruitment clusters of health centres randomised to the 2+1 schedule and those in recruitment clusters of health centres

For peer review only - http://bmjopen.bmj.com/site/about/guidelines.xhtml

randomised to the 3+0 schedule. Indirect effects will be evidenced by comparing children in the recruitment clusters of health centres randomised to the 3+0 schedule and older children (5-10 years) and HIV+ adults. Additionally, data obtained in this study will be compared to those obtained from previous carriage surveys to ascertain any changes in VT carriage prevalence before and after the PVC13 schedule change. Statistical tests will be selected depending on the distribution patterns of the data. Potential confounders and sources if interaction (including age, gender and health centre) will be identified by testing the association between variables and VT carriage and included in the multivariable models when p<0·1. Sensitivity analyses will include assessing impact of i) receiving only one, only two, or all three doses PCV; ii) having document-confirmed PCV vaccination or iii) schedule adherence to within 2 weeks of each scheduled dose on VT prevalence and on the VT distribution.

Ethics and Dissemination

Ethics Approvals. The study has been approved by the Malawi College of Medicine Research Ethics Committee (COMREC; Ref: P05.19.2680), the University College London Research Ethics Committee (Ref: 8603.002) and the University of Liverpool Research Ethics Committee (Ref: 5439).

Data Monitoring External Advisory Group. The implementation of the study protocol will be reviewed and monitored by an External Advisory Group, providing oversight of the study activities and advise the study team at the time of the interim and final analyses. The group will include experts from the University of Malawi, College of Medicine, the London School of Hygiene & Tropical Medicine, and the Medical Research Council.

Interim Analysis and Changes to Public Health Policy. Upon completion of the first carriage survey, an interim analysis will be implemented using data obtained on the VT carriage prevalence among vaccinated children 15-24 months of age. These results will be used to assess possible adaptation of the second community carriage survey. Three possible scenarios are considered: i) If convincing evidence of major change in carriage prevalence is demonstrated, immediate action will be discussed with the MoH to move to change the schedule and adapt year-3 sampling; ii) If no

For peer review only - http://bmjopen.bmj.com/site/about/guidelines.xhtml

major change in carriage prevalence is identified, the study will continue as planned; iii) If carriage prevalence has fallen by more than 30% but does not meet the primary threshold of 50%, the second carriage survey will be brought forward by 6 months. Decisions will be reached in consensus between investigators, the Expert Advisory Board, and the MoH.

Informed Consent Process. The study will only recruit children whose parents/legal guardians have the capacity to provide informed consent or, in the case of adults, are capable of giving consent. Children who are minors but ≥8 years of age will be required to provide informed assent, in addition to parents/legal guardians providing informed consent. Participants will receive both verbal and written information about the study and will be given the opportunity to ask questions and express their doubts and concerns before accepting to take part. They will also be given time to reflect before they come to a decision. An informed consent and/or assent form will be signed and dated by the participant and a member of the research team. The participant will keep a copy of the document, and a second one will be kept in the study file with the Principal Investigator based in Blantyre, Malawi. Participants will be informed of their right to withdraw consent at any point until study ends without the need to provide a reason, and without penalty.

For peer review only

Dissemination Policy and Plans

Study results will be shared with local stakeholders and published in peer-reviewed journals. Partial results and interim analyses will be shared with the Malawi MoH and other relevant policymakers and decision-making stakeholders. Partial and final findings will be presented at relevant international conferences and meetings. Copies of all published materials and reports will be shared with the research ethics committees (RECs) and collaborators. We will return to the community partners and work with the community advisory board to report and further disseminate our results into those communities where we worked.

Data availability. Data used in analyses for reported findings of this study will be made publicly available on FigShare (https://figshare.com), an online open access repository.

For peer review only - http://bmjopen.bmj.com/site/about/guidelines.xhtml

Acknowledgements. The authors thank the MLW laboratory management team, led by Brigitte Denis and George Selemani.

For peer review only

For peer review only - http://bmjopen.bmj.com/site/about/guidelines.xhtml

References

1. Heinsbroek E, Tafatatha T, Phiri A, Swarthout TD, Alaerts M, Crampin AC, et al. Pneumococcal carriage in households in Karonga District, Malawi, before and after introduction of 13-valent pneumococcal conjugate vaccination. Vaccine. 2018 Oct 20;

2. GBD 2016 Lower Respiratory Infections Collaborators. Estimates of the global, regional, and national morbidity, mortality, and aetiologies of lower respiratory infections in 195 countries, 1990-2016: a systematic analysis for the Global Burden of Disease Study 2016. Lancet Infect Dis. 2018;18(11):1191–210.

3. Gray BM, Turner ME, Dillon HC. Epidemiologic studies of Streptococcus pneumoniae in infants. The effects of season and age on pneumococcal acquisition and carriage in the first 24 months of life. Am J Epidemiol. 1982 Oct;116(4):692–703.

4. Ghaffar F, Friedland IR, McCracken GH. Dynamics of nasopharyngeal colonization by Streptococcus pneumoniae. Pediatr Infect Dis J. 1999 Jul;18(7):638–46.

5. Högberg L, Geli P, Ringberg H, Melander E, Lipsitch M, Ekdahl K. Age- and Serogroup-Related Differences in Observed Durations of Nasopharyngeal Carriage of Penicillin-Resistant Pneumococci. J Clin Microbiol. 2007 Mar 1;45(3):948–52.

6. Usuf E, Bottomley C, Adegbola RA, Hall A. Pneumococcal carriage in sub-Saharan Africa--a systematic review. PloS One. 2014;9(1):e85001.

7. Vallès X, Flannery B, Roca A, Mandomando I, Sigaúque B, Sanz S, et al. Serotype distribution and antibiotic susceptibility of invasive and nasopharyngeal isolates of Streptococcus pneumoniae among children in rural Mozambique. Trop Med Int Health TM IH. 2006 Mar;11(3):358–66.

8. Hill PC, Townend J, Antonio M, Akisanya B, Ebruke C, Lahai G, et al. Transmission of Streptococcus pneumoniae in rural Gambian villages: a longitudinal study. Clin Infect Dis Off Publ Infect Dis Soc Am. 2010 Jun 1;50(11):1468–76.

9. Abdullahi O, Karani A, Tigoi CC, Mugo D, Kungu S, Wanjiru E, et al. Rates of acquisition and clearance of pneumococcal serotypes in the nasopharynges of children in Kilifi District, Kenya. J Infect Dis. 2012 Oct 1;206(7):1020–9.

10. Mackenzie GA, Hill PC, Jeffries DJ, Hossain I, Uchendu U, Ameh D, et al. Effect of the introduction of pneumococcal conjugate vaccination on invasive pneumococcal disease in The Gambia: a population-based surveillance study. Lancet Infect Dis. 2016;16(6):703–11.

11. von Gottberg A, de Gouveia L, Tempia S, Quan V, Meiring S, von Mollendorf C, et al. Effects of Vaccination on Invasive Pneumococcal Disease in South Africa. N Engl J Med. 2014 Nov 13;371(20):1889–99.

For peer review only

For peer review only - http://bmjopen.bmj.com/site/about/guidelines.xhtml

12. Hammitt LL, Etyang AO, Morpeth SC, Ojal J, Mutuku A, Mturi N, et al. Effect of ten-valent pneumococcal conjugate vaccine on invasive pneumococcal disease and nasopharyngeal carriage in Kenya: a longitudinal surveillance study. The Lancet. 2019 May 25;393(10186):2146–54.

13. Roca A, Bojang A, Bottomley C, Gladstone RA, Adetifa JU, Egere U, et al. Effect on nasopharyngeal pneumococcal carriage of replacing PCV7 with PCV13 in the Expanded Programme of Immunization in The Gambia. Vaccine. 2015 Dec 16;33(51):7144–51.

14. Adebanjo T, Lessa FC, Mucavele H, Moiane B, Chauque A, Pimenta F, et al. Pneumococcal carriage and serotype distribution among children with and without pneumonia in Mozambique, 2014-2016. PloS One. 2018;13(6):e0199363.

15. Flasche S, Van Hoek AJ, Sheasby E, Waight P, Andrews N, Sheppard C, et al. Effect of pneumococcal conjugate vaccination on serotype-specific carriage and invasive disease in England: a cross-sectional study. PLoS Med. 2011 Apr;8(4):e1001017.

16. Jayasinghe S, Menzies R, Chiu C, Toms C, Blyth CC, Krause V, et al. Long-term Impact of a “3 + 0” Schedule for 7- and 13-Valent Pneumococcal Conjugate Vaccines on Invasive Pneumococcal Disease in Australia, 2002–2014. Clin Infect Dis. 2017 Jan 15;64(2):175–83.

17. Moore MR, Link-Gelles R, Schaffner W, Lynfield R, Lexau C, Bennett NM, et al. Effect of use of 13-valent pneumococcal conjugate vaccine in children on invasive pneumococcal disease in children and adults in the USA: analysis of multisite, population-based surveillance. Lancet Infect Dis. 2015 Mar;15(3):301–9.

18. Prato R, Fortunato F, Cappelli MG, Chironna M, Martinelli D. Effectiveness of the 13-valent pneumococcal conjugate vaccine against adult pneumonia in Italy: a case-control study in a 2-year prospective cohort. BMJ Open. 2018 25;8(3):e019034.

19. Rinta-Kokko H, Palmu AA, Auranen K, Nuorti JP, Toropainen M, Siira L, et al. Long-term impact of 10-valent pneumococcal conjugate vaccination on invasive pneumococcal disease among children in Finland. Vaccine. 2018 05;36(15):1934–40.

20. Ruiz-Contreras J, Picazo J, Casado-Flores J, Baquero-Artigao F, Hernández-Sampelayo T, Otheo E, et al. Impact of 13-valent pneumococcal conjugate vaccine on pneumococcal meningitis in children. Vaccine. 2017 Aug 16;35(35, Part B):4646–51.

21. Waight PA, Andrews NJ, Ladhani SN, Sheppard CL, Slack MPE, Miller E. Effect of the 13-valent pneumococcal conjugate vaccine on invasive pneumococcal disease in England and Wales 4 years after its introduction: an observational cohort study. Lancet Infect Dis. 2015 May;15(5):535–43.

For peer review only - http://bmjopen.bmj.com/site/about/guidelines.xhtml

22. Wiese AD, Griffin MR, Grijalva CG. Impact of pneumococcal conjugate vaccines on hospitalizations for pneumonia in the United States. Expert Rev Vaccines. 2019 Feb 13;0(ja):null.

23. Wiese AD, Huang X, Yu C, Mitchel EF, Kyaw MH, Griffin MR, et al. Changes in otitis media episodes and pressure equalization tube insertions among young children following introduction of the 13-valent pneumococcal conjugate vaccine: A birth-cohort based study. Clin Infect Dis Off Publ Infect Dis Soc Am. 2019 Feb 16;

24. Miller E, Andrews NJ, Waight PA, Slack MP, George RC. Herd immunity and serotype replacement 4 years after seven-valent pneumococcal conjugate vaccination in England and Wales: an observational cohort study. Lancet Infect Dis. 2011 Oct;11(10):760–8.

25. O'Brien KL, Dagan R. The potential indirect effect of conjugate pneumococcal vaccines. Vaccine. 2003 May 16;21(17–18):1815–25.

26. Whitney CG, Farley MM, Hadler J, Harrison LH, Bennett NM, Lynfield R, et al. Decline in invasive pneumococcal disease after the introduction of protein-polysaccharide conjugate vaccine. N Engl J Med. 2003 May 1;348(18):1737–46.

27. Melegaro A, Edmunds WJ. Cost-effectiveness analysis of pneumococcal conjugate vaccination in England and Wales. Vaccine. 2004 Oct 22;22(31–32):4203–14.

28. Simell B, Auranen K, Käyhty H, Goldblatt D, Dagan R, O'Brien KL, et al. The fundamental link between pneumococcal carriage and disease. Expert Rev Vaccines. 2012 Jul;11(7):841–55.

29. Auranen K, Rinta-Kokko H, Goldblatt D, Nohynek H, O'Brien KL, Satzke C, et al. Colonisation endpoints in Streptococcus pneumoniae vaccine trials. Vaccine. 2013 Dec 17;32(1):153–8.

30. Kobayashi M, Conklin LM, Bigogo G, Jagero G, Hampton L, Fleming-Dutra KE, et al. Pneumococcal carriage and antibiotic susceptibility patterns from two cross-sectional colonization surveys among children aged <5 years prior to the introduction of 10-valent pneumococcal conjugate vaccine - Kenya, 2009-2010. BMC Infect Dis. 2017 05;17(1):25.

31. Nunes MC, Shiri T, van Niekerk N, Cutland CL, Groome MJ, Koen A, et al. Acquisition of Streptococcus pneumoniae in pneumococcal conjugate vaccine-naïve South African children and their mothers. Pediatr Infect Dis J. 2013 May;32(5):e192-205.

32. Usuf E, Badji H, Bojang A, Jarju S, Ikumapayi UN, Antonio M, et al. Pneumococcal carriage in rural Gambia prior to the introduction of pneumococcal conjugate vaccine: a population-based survey. Trop Med Int Health TM IH. 2015 Jul;20(7):871–9.

33. Verani JR, Massora S, Acácio S, Santos RT dos, Vubil D, Pimenta F, et al. Nasopharyngeal carriage of Streptococcus pneumoniae among HIV-infected

For peer review only - http://bmjopen.bmj.com/site/about/guidelines.xhtml

and –uninfected children <5 years of age before introduction of pneumococcal conjugate vaccine in Mozambique. PLOS ONE. 2018 Feb 15;13(2):e0191113.

34. Bar-Zeev N, Swarthout TD, Everett DB, Alaerts M, Msefula J, Brown C, et al. Impact and Effectiveness of 13-Valent Pneumococcal Conjugate Vaccine on Population Incidence of Vaccine and Non-Vaccine Serotype Invasive Pneumococcal Disease in Blantyre, Malawi, 2006-2018: Prospective Observational Time-Series and Case-Control Studies [Internet]. Rochester, NY: Social Science Research Network; 2020 Dec [cited 2021 Jan 27]. Report No.: ID 3745169. Available from: https://papers.ssrn.com/abstract=3745169

35. King C, Bar-Zeev N, Phiri T, Beard J, Mvula H, Crampin A, et al. Population impact and effectiveness of sequential 13-valent pneumococcal conjugate and monovalent rotavirus vaccine introduction on infant mortality: prospective birth cohort studies from Malawi. BMJ Glob Health. 2020 Sep;5(9).

36. Cutts FT, Zaman SMA, Enwere G, Jaffar S, Levine OS, Okoko JB, et al. Efficacy of nine-valent pneumococcal conjugate vaccine against pneumonia and invasive pneumococcal disease in The Gambia: randomised, double-blind, placebo-controlled trial. Lancet Lond Engl. 2005 Apr 26;365(9465):1139–46.

37. Kwambana-Adams B, Hanson B, Worwui A, Agbla S, Foster-Nyarko E, Ceesay F, et al. Rapid replacement by non-vaccine pneumococcal serotypes may mitigate the impact of the pneumococcal conjugate vaccine on nasopharyngeal bacterial ecology. Sci Rep. 2017 15;7(1):8127.

38. Usuf E, Bottomley C, Bojang E, Cox I, Bojang A, Gladstone R, et al. Persistence of nasopharyngeal pneumococcal vaccine serotypes and increase of non-vaccine serotypes among vaccinated infants and their mothers five years after PCV13 introduction in The Gambia. Clin Infect Dis Off Publ Infect Dis Soc Am. 2018 Aug 24;

39. WHO Publication. Pneumococcal vaccines WHO position paper - 2012 - recommendations. Vaccine. 2012 Jul 6;30(32):4717–8.

40. Fleming-Dutra KE, Conklin L, Loo JD, Knoll MD, Park DE, Kirk J, et al. Systematic review of the effect of pneumococcal conjugate vaccine dosing schedules on vaccine-type nasopharyngeal carriage. Pediatr Infect Dis J. 2014 Jan;33 Suppl 2:S152-160.

41. Andrejko K, Hosangadi D, Cohen O, Cohen AL, Althouse B, McIntyre P, et al. WHO Technical Expert Consultation Report on Optimization of PCV Impact: Review of Evidence and Programmatic Considerations to Inform Policy. 2017;29.

42. World Health Organization. Pneumococcal Conjugate Vaccine (PCV) Review of Impact Evidence (PRIME) Summary of Findings from Systematic Review [Internet]. 2017 Oct [cited 2019 Mar 12] p. 215. Available from: https://www.who.int/immunization/sage/meetings/2017/october/3_FULL_PRIME_REPORT_2017Sep26.pdf?ua=1

For peer review only

For peer review only - http://bmjopen.bmj.com/site/about/guidelines.xhtml

43. Mvula H, Heinsbroek E, Chihana M, Crampin AC, Kabuluzi S, Chirwa G, et al. Predictors of Uptake and Timeliness of Newly Introduced Pneumococcal and Rotavirus Vaccines, and of Measles Vaccine in Rural Malawi: A Population Cohort Study. PLoS ONE [Internet]. 2016 May 6 [cited 2019 Mar 12];11(5). Available from: https://www.ncbi.nlm.nih.gov/pmc/articles/PMC4859501/

44. Tsega A, Hausi H, Chriwa G, Steinglass R, Smith D, Valle M. Vaccination coverage and timely vaccination with valid doses in Malawi. Vaccine Rep. 2016 Dec 1;6:8–12.

45. Swarthout TD, Fronterre C, Lourenço J, Obolski U, Gori A, Bar-Zeev N, et al. High residual carriage of vaccine-serotype Streptococcus pneumoniae after introduction of pneumococcal conjugate vaccine in Malawi. Nat Commun. 2020 May 6;11(1):2222.

46. Lourenço J, Obolski U, Swarthout TD, Gori A, Bar-Zeev N, Everett D, et al. Determinants of high residual post-PCV13 pneumococcal vaccine-type carriage in Blantyre, Malawi: a modelling study. BMC Med. 2019 Dec 5;17(1):219.

47. National Statistical Office. Government of Malawi. 2018 Malawi Population and Housing Census Main Report [Internet]. Zomba. Malawi: Government of Malawi; 2019 [cited 2019 Nov 8]. Available from: https://malawi.unfpa.org/sites/default/files/resource-pdf/2018%20Malawi%20Population%20and%20Housing%20Census%20Main%20Report%20%281%29.pdf

48. Loudon K, Treweek S, Sullivan F, Donnan P, Thorpe KE, Zwarenstein M. The PRECIS-2 tool: designing trials that are fit for purpose. BMJ. 2015 May 8;350:h2147.

49. Satzke C, Turner P, Virolainen-Julkunen A, Adrian PV, Antonio M, Hare KM, et al. Standard method for detecting upper respiratory carriage of Streptococcus pneumoniae: updated recommendations from the World Health Organization Pneumococcal Carriage Working Group. Vaccine. 2013 Dec 17;32(1):165–79.

Author's contribution: Contributors TDS, GK, CM, NF, and RHS conceived the study. TDS, GK, CM, NF, and RHS designed the study with contributions from AI, JC, JK, MB, MYRH, and SBG. TDS, CB and JM oversaw development of laboratory methods. TDS, AI, AG, PC, FB, RN, EB and GS designed study documents. TDS and AI jointly wrote the first draft. All authors have commented on the manuscript. All authors have read and approved the final manuscript.

Competing interest. The authors declare that they have no competing interests.

For peer review only - http://bmjopen.bmj.com/site/about/guidelines.xhtml

646 **Funding** This work was funded by Bill & Melinda Gates Foundation, USA (Ref: OPP1185516) to RSH; National Institute for Health Research (NIHR) Global Health Research Unit on Mucosal Pathogens using UK aid from the UK Government (Project number 16/136/46) to RSH. The MLW Clinical Research Programme is supported by a Strategic Award from the Wellcome Trust, UK (206545/Z/17/Z) to SG. The funders had no role in study design. The funders will have no role in collection, analysis, data interpretation, writing of the report or in the decision to submit the paper for publication. The corresponding author will have full access to the study data and, together with the senior authors, will have final responsibility for the decision to submit for publication.

For peer review only

For peer review only - http://bmjopen.bmj.com/site/about/guidelines.xhtml

Figure Legends

Figure 1. Map of Blantyre District with borders of health centre catchment areas Red crosses (+) indicate location of health centres offering EPI vaccination. Areas shaded in green are non-inhabited (incl. mountains, industrial zones, and other regions administratively declared not for habitation).

Figure 2. The PRagmatic-Explanatory Continuum Indicator Summary 2 (PRECIS-2) wheel with study-specific scores.

Figure 3. Carriage surveillance sampling frame Black dashed vertical line separates primary (left of dashed line) from secondary study objectives. QECH=Queen Elizabeth Central Hospital, PCV=pneumococcal conjugate vaccine, ART=anti-retroviral, m=months, yrs=years

For peer review only

For peer review only - http://bmjopen.bmj.com/site/about/guidelines.xhtml

670 **Table 1:** Sample size estimations

N per cluster	ICC	p1	p2	power	alpha	DE	N per arm	Clusters, per arm	Clusters, total	N total
40	0.005	0.14	0.07	0.8	0.05	1.20	358	9	18	720
40	0.005	0.14	0.07	0.8	0.05	1.21	400	10	20	800
45	0.005	0.14	0.07	0.8	0.05	1.22	366	9	18	810
50	0.005	0.14	0.07	0.8	0.05	1.25	373	8	16	800
55	0.005	0.14	0.07	0.8	0.05	1.27	381	7	14	770
60	0.005	0.14	0.07	0.8	0.05	1.30	388	7	14	840
40	0.01	0.14	0.07	0.8	0.05	1.39	417	11	22	880
45	0.01	0.14	0.07	0.8	0.05	1.44	432	10	20	900
50	0.01	0.14	0.07	0.8	0.05	1.49	447	9	18	900
55	0.01	0.14	0.07	0.8	0.05	1.54	462	9	18	990
60	0.01	0.14	0.07	0.8	0.05	1.59	477	8	16	960
40	0.015	0.14	0.07	0.8	0.05	1.59	475	12	24	960

671 DE=design effect, N=number of children recruited, ICC=intra-class correlation

For peer review only

For peer review only - http://bmjopen.bmj.com/site/about/guidelines.xhtml

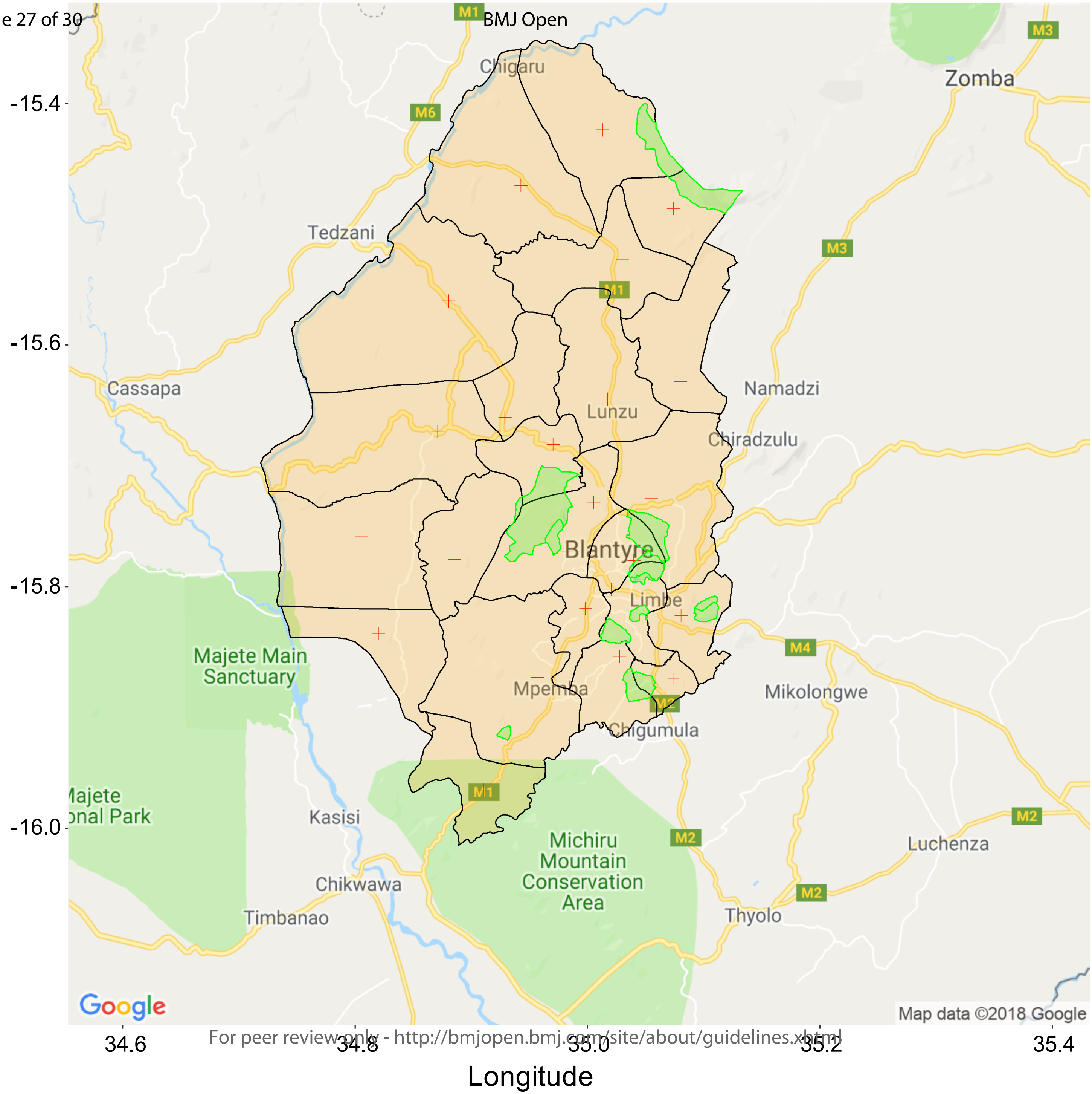

-15.4
-15.6
-15.8
-16.0
Latitude
34.6
34.8
35.0
35.2
35.4
Longitude
Chigaru
Zomba
Tedzani
Cassapa
Namadzi
Lunzu
Chiradzulu
Blantyre
Limbe
Majete Main Sanctuary
Mikolongwe
Mpemba
Chigumula
Kasisi
Michiru Mountain Conservation Area
Luchenza
Chikwawa
Timbanao
Thyolo
M1
M3
M6
M4
M2
Google
Map data ©2018 Google

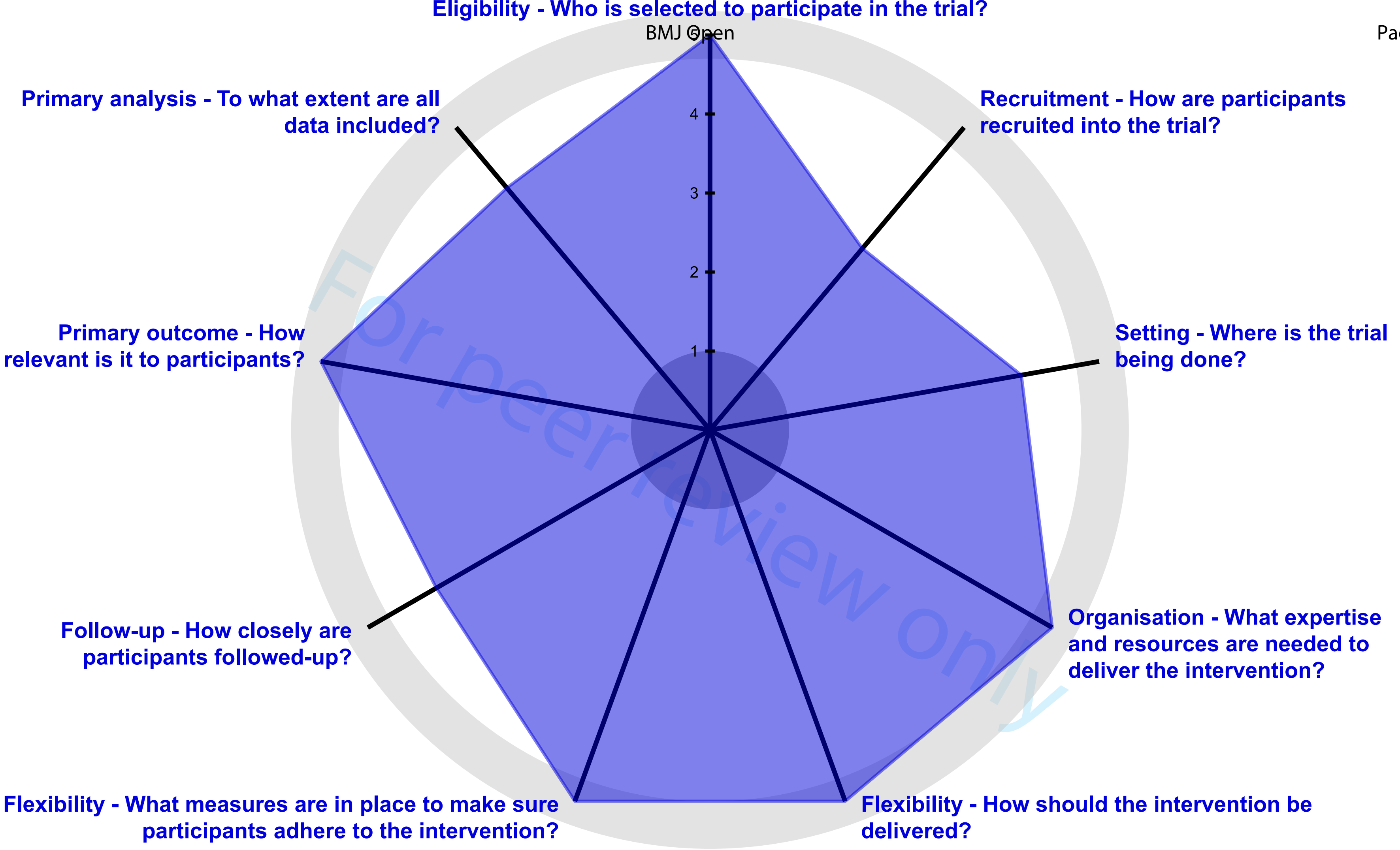

Eligibility	Recruitment	Setting	Organisation	Flexibility (delivery)	Flexibility (Adhere)	Follow-up	Primary outcome	Primary analysis
5	3	4	5	5	5	4	5	4

For peer review only - http://bmjopen.bmj.com/site/about/guidelines.xhtml

Surveillance Sampling Frame

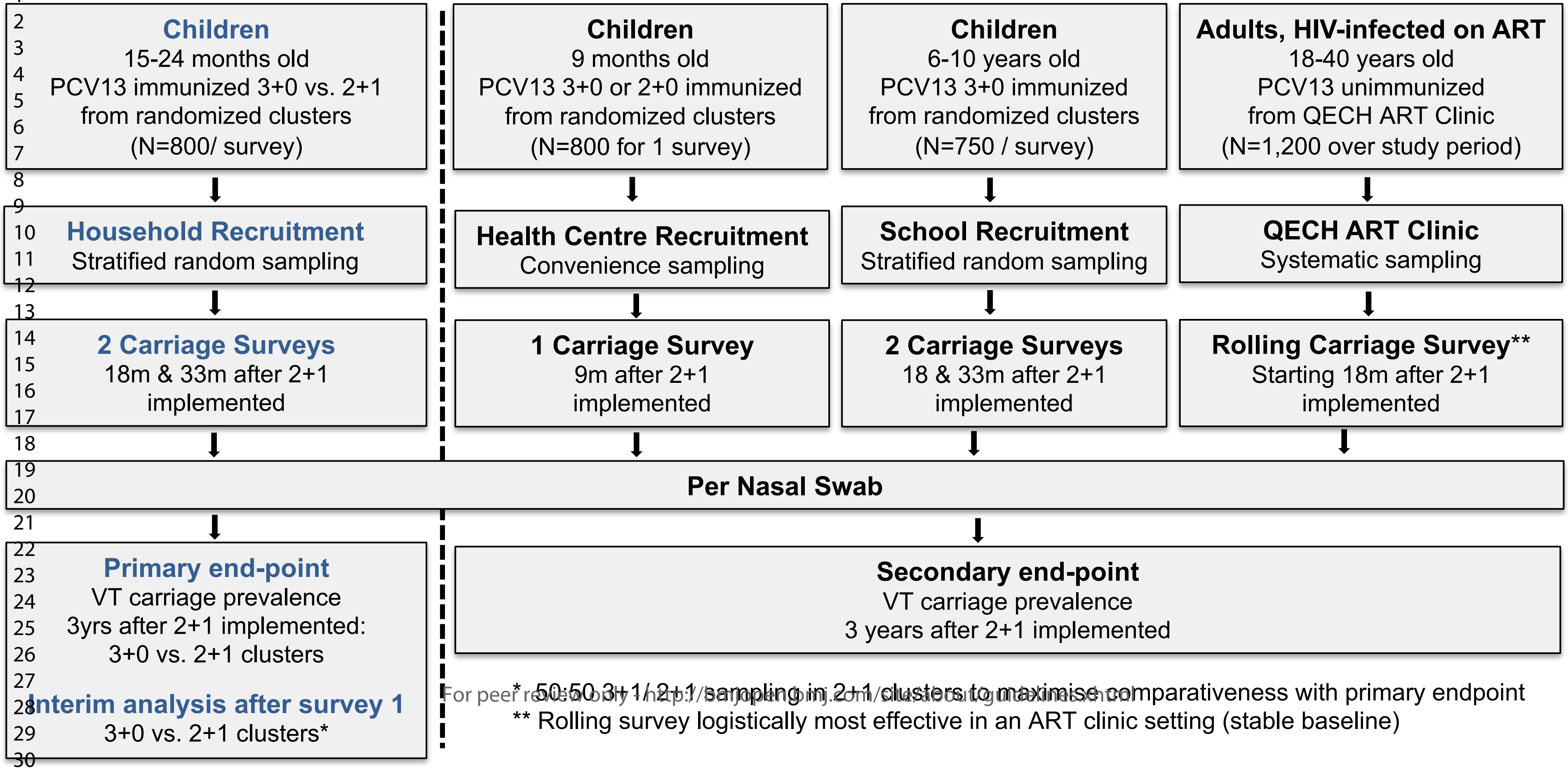

STROBE Statement—Checklist of items that should be included in reports of ***cross-sectional studies***

	Item No	Recommendation	Page in Protocol
**Title and abstract**	1	(*a*) Indicate the study's design with a commonly used term in the title or the abstract	Title: Page 1<br>Abstract: Page 2, lines 13-14
		(*b*) Provide in the abstract an informative and balanced summary of what was done and what was found	What will be done:<br>Page 2, lines 14-21<br><br>What will be found:<br>Page2, lines 22-24
**Introduction**			
Background/rationale	2	Explain the scientific background and rationale for the investigation being reported	Page 5, line 103-116<br>Page 6, lines 132-134
Objectives	3	State specific objectives, including any prespecified hypotheses	Introduction:<br>Page 6, lines 137-140<br><br>Methods:<br>Page 7, line 169-181
**Methods**			
Study design	4	Present key elements of study design early in the paper	Page 7, line 142-145
Setting	5	Describe the setting, locations, and relevant dates, including periods of recruitment, exposure, follow-up, and data collection	Page 6, lines 134-140<br>Page 7, line 147-155<br>Page 9, lines 226-230
Participants	6	(*a*) Give the eligibility criteria, and the sources and methods of selection of participants	Participant eligibility:<br>Page 11, lines 277-301<br><br>Participant selection:<br>Page 9, lines 236-246<br>Page 10, lines 253-257<br>Page 10, lines 263-264
Variables	7	Clearly define all outcomes, exposures, predictors, potential confounders, and effect modifiers. Give diagnostic criteria, if applicable	Page 12, lines 314-325<br>Page 14, line 381-386
Data sources/ measurement	8*	For each variable of interest, give sources of data and details of methods of assessment (measurement). Describe comparability of assessment methods if there is more than one group	Vaccine schedule received:<br>Page 8, line 198-201<br><br>Vaccination status:<br>Page 9, line 233-234
Bias	9	Describe any efforts to address potential sources of bias	Page 8, lines 183-188<br>Page 9 line 236-246<br>Page 10, lines 254-257
Study size	10	Explain how the study size was arrived at	Page 12, lines 336-344
Quantitative variables	11	Explain how quantitative variables were handled in the analyses. If applicable, describe which groupings were	Page 13, lines 369-380

For peer review only - http://bmjopen.bmj.com/site/about/guidelines.xhtml

		chosen and why	Groupings:<br>Page 9, lines 233-236<br>Page 10, lines 248-253<br>Page 10, lines 259-263
Statistical methods	12	(*a*) Describe all statistical methods, including those used to control for confounding	Page 12, line 369-386
		(*b*) Describe any methods used to examine subgroups and interactions	Page 14, line 381-383
		(*c*) Explain how missing data were addressed	Page 13, lines 361-366
		(*d*) If applicable, describe analytical methods taking account of sampling strategy	Not applicable
		(*e*) Describe any sensitivity analyses	Page 14, line 383-386
**Results**			
Participants	13*	(a) Report numbers of individuals at each stage of study—eg numbers potentially eligible, examined for eligibility, confirmed eligible, included in the study, completing follow-up, and analysed	Not applicable for a protocol manuscript
		(b) Give reasons for non-participation at each stage	Not applicable for a protocol manuscript
		(c) Consider use of a flow diagram	Not applicable for a protocol manuscript - Will be included in post-implementation manuscript
Descriptive data	14*	(a) Give characteristics of study participants (eg demographic, clinical, social) and information on exposures and potential confounders	Not applicable for a protocol manuscript
		(b) Indicate number of participants with missing data for each variable of interest	Not applicable for a protocol manuscript
Outcome data	15*	Report numbers of outcome events or summary measures	Not applicable for a protocol manuscript
Main results	16	(*a*) Give unadjusted estimates and, if applicable, confounder-adjusted estimates and their precision (eg, 95% confidence interval). Make clear which confounders were adjusted for and why they were included	Not applicable for a protocol manuscript
		(*b*) Report category boundaries when continuous variables were categorized	Not applicable for a protocol manuscript
		(*c*) If relevant, consider translating estimates of relative risk into absolute risk for a meaningful time period	Not applicable for a protocol manuscript
Other analyses	17	Report other analyses done—eg analyses of subgroups and interactions, and sensitivity analyses	Not applicable for a protocol manuscript
**Discussion**			
Key results	18	Summarise key results with reference to study objectives	Not applicable for a protocol manuscript
Limitations	19	Discuss limitations of the study, taking into account sources of potential bias or imprecision. Discuss both direction and magnitude of any potential bias	Not applicable for a protocol manuscript

For peer review only

Interpretation	20	Give a cautious overall interpretation of results considering objectives, limitations, multiplicity of analyses, results from similar studies, and other relevant evidence	Not applicable for a protocol manuscript
Generalisability	21	Discuss the generalisability (external validity) of the study results	Not applicable for a protocol manuscript
**Other information**			
Funding	22	Give the source of funding and the role of the funders for the present study and, if applicable, for the original study on which the present article is based	Page 22, lines 646-654

*Give information separately for exposed and unexposed groups.

Note: An Explanation and Elaboration article discusses each checklist item and gives methodological background and published examples of transparent reporting. The STROBE checklist is best used in conjunction with this article (freely available on the Web sites of PLoS Medicine at http://www.plosmedicine.org/, Annals of Internal Medicine at http://www.annals.org/, and Epidemiology at http://www.epidem.com/). Information on the STROBE Initiative is available at www.strobe-statement.org.

For peer review only

For peer review only - http://bmjopen.bmj.com/site/about/guidelines.xhtml
