# Supplementary material for: A pragmatic health centre-based evaluation comparing the effectiveness of a PCV13 schedule change from 3+0 to 2+1 in a high pneumococcal carriage and disease burden setting in Malawi: a study protocol
Source: BMJ Open. 2021 Jun 17;11(6):e050312. doi: 10.1136/bmjopen-2021-050312 (PMC8212416; doi:10.1136/bmjopen-2021-050312)
Supplement: Reviewer comments [file bmjopen-2021-050312.reviewer_comments.pdf]

PEER REVIEW HISTORY

BMJ Open publishes all reviews undertaken for accepted manuscripts. Reviewers are asked to complete a checklist review form (**http://bmjopen.bmj.com/site/about/resources/checklist.pdf**) and are provided with free text boxes to elaborate on their assessment. These free text comments are reproduced below.

ARTICLE DETAILS

**TITLE (PROVISIONAL)**	A pragmatic health centre-based evaluation comparing the effectiveness of a PCV13 schedule change from 3+0 to 2+1 in a high pneumococcal carriage and disease burden setting in Malawi: a study protocol
**AUTHORS**	Swarthout, Todd; Ibarz-Pavon, Ana; Kawalazira, Gift; Sinjani, George; Chirombo, James; Gori, Andrea; Chalusa, Peter; Bonomali, Farouck; Nyirenda, Roseline; Bulla, Edwin; Brown, Comfort; Msefula, Jacquline; Banda, Marjory; Kachala, Jean; Mwansambo, Charles; Henrion, Marc; Gordon, Stephen; French, Neil; Heyderman, Robert

This article was not externally reviewed at BMJ Open. Protocols that have been independently assessed prior to submission to BMJ Open are usually fast-tracked to publication on the grounds that further substantial changes will not be possible. This independent assessment will usually be external, independent review for both a substantial grant award from a non-commercial or government funder and ethics approval.
